# Legal Weakness, Investment Risks, and Distressed Acquisitions: Evidence from Russian Regions

**DOI:** 10.1057/s41294-022-00203-5

**Published:** 2022-12-14

**Authors:** Ichiro Iwasaki, Yuko Adachi

**Affiliations:** 1 Russian Research Center, Institute of Economic Research, Hitotsubashi University, Tokyo, Japan; 2 Faculty of Foreign Studies, Sophia University, Tokyo, Japan

**Keywords:** Legal weakness, Investment risk, Financial distress, Distressed acquisitions, Russia, C35, D02, D22, E02, G34, K20, L22

## Abstract

This paper traces the survival status of 93,260 Russian business firms in the period of 2007–2019 and empirically examines the determinants of the acquisition of financially distressed companies (i.e., distressed acquisitions). We found that, of 93,260 firms, 50,743 failed in management, and among these distressed firms, 10,110 were rescued by acquisition during the observation period. Our empirical results indicate that, in Russian regions, the weakness of the legal system tends to increase the probability of distressed acquisitions, while other socioeconomic risks negatively affect it. These tendencies are common in most industries and regions. It is also revealed that, in the most-developed area, monotown enterprises are more likely than other firms to be bailed out by acquisition after management failure, but it is not always true for the whole federation.

## Introduction

The days when Russia attracted the attention of investors around the world as a fast-growing emerging market are now long gone. Citizens and companies in the country have had to endure a series of hardships, beginning with the Lehman shock of 2008 and continuing with the COVID-19 pandemic crisis. Contrary to the initial expectation of the International Monetary Fund (IMF) and the Federal Government of Russia, the negative impact of the new coronavirus infection on the Russian economy was not comparable to that of the 2008 global financial crisis,^1^ but the real GDP growth rate in 2020 has sunk to minus 2.7% anyway, undoubtedly increasing the economic difficulties in the country more than ever in the last two decades (Iwasaki 2022). Although economic growth in 2021 appears to be positive at nearly 5%, it is highly likely that, in 2022, the war with Ukraine and the unprecedented economic sanctions imposed by the international community will plunge Russia into a deep recession (World Bank 2022).

**Fig. 1:**
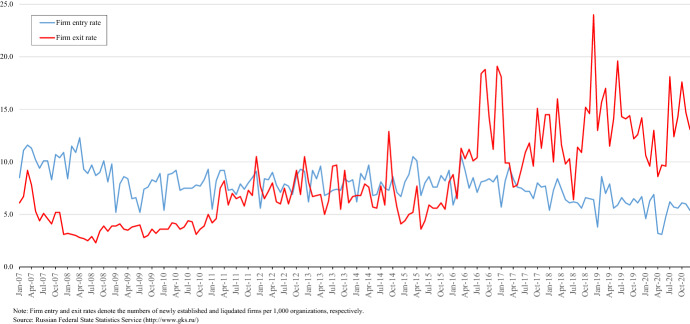
Dynamics of firm entry and exit in Russia: 2007–2020

The dynamics of firm entry and exit well reflect the painful path of the Russian economy. In fact, as shown in Fig. 1, the firm entry rate has recorded a long slump after the 2008 crisis until the end of 2020. During the same period, the firm exit rate continued to rise steadily. To make matters worse, since 2016, the exit rate has almost always been higher than the entry rate; as a result, according to the Federal State Statistic Service (Rosstat), the total number of business companies and organizations declined from 4,507,000 in January 2007 to 3,827,000 in January 2020, meaning that a net of 15.1% of Russian firms were lost during these 14 years. Even with accounting for the trend of an aging population, there is no doubt that the vitality of the Russian business sector has been seriously impaired.

Russia is known as a country of active mergers and acquisitions. In this country, hostile takeovers frequently occur, and many Russian managers are frightened by the risk (Rochliz 2014; Frye 2017). As reported later, however, mergers and acquisitions (M&A) are also intensively used to rescue companies that have fallen into financial distress. When the legal system is unreliable, so-called “distressed acquisitions” are used as an alternative to the legal treatment of debts and assets of failed companies; therefore, the opportunity cost of company liquidation in accordance with the law is higher than that of acquisition. In this sense, distressed acquisitions in Russia were likely to function as a complementary mechanism to the weaknesses of the legal system in the period of economic transition. However, in light of the above-mentioned facts about company demographics, there may be significant changes in the role of distressed acquisitions in recent years.

Consistent with the arguments above, Iwasaki et al. (2021) demonstrated that the quality and enforcement of insolvency laws are negatively associated with the probability of distressed acquisitions in European emerging markets, including Russia. In other words, they found that national-level institutional quality can effectively explain the differences in the frequency of distressed acquisitions across Eastern Europe. As we will report later, however, the frequency of acquisition of failed firms varies markedly across Russian regions. The empirical framework of Iwasaki et al. (2021) is not capable of explaining this phenomenon. Firms’ institutional and other management environments have a multilayered structure from the national to the regional level. Therefore, in order to fully elucidate the determinants of distressed acquisitions in a country, the perspective of empirical analysis should be directed not only to national-level factors but also to those in regions. In this paper, we expand on the findings of Iwasaki et al. (2021) by addressing this issue.

Furthermore, in their empirical analysis, Iwasaki et al. (2021) regressed the probability of distressed acquisitions in 17 European emerging markets during the period of 2007–2019 on a series of firm-level attributes and the national-level institutional quality observed in 2006, proving the high predictability of the initial conditions on the probability of distressed acquisitions. While the empirical method employed by Iwasaki et al. (2021) is similar to a survival analysis and is, thus, effective in avoiding or significantly mitigating the issue of potential endogeneity,^2^ it raises the question of whether their finding that initial conditions remain effective over a decade can be replicated. In this paper, we question whether the empirical approach of Iwasaki et al. (2021) is valid even if we restrict our target country to Russia and use institutional quality variables at the regional rather than the national level.

To this end, using a large dataset of Russian business firms in the period of 2007–2019, we first attempt to estimate the frequency of acquisition of financially distressed companies and grasp its time trend. Then, following the empirical strategy of Iwasaki et al. (2021), we empirically examine the determinants of distressed acquisitions with a special focus on the initial conditions of Russian regions, including not only the quality of the legal system but also socioeconomic investment risks and the socialist legacy—monotowns (*monogorody*). For reasons discussed later, we expect the latter two factors to have as much influence on the acquisition of failed firms in Russia as the former. Through empirical testing of this assumption, we provide new insights into the literature.

Of 93,260 Russian firms, we found 50,743 to be financially distressed, and among these failed firms, 10,110 were bailed out by acquisition in the period of 2007–2019. We also found that the share of distressed acquisitions of failed firms fell sharply during the observation period. Our empirical results indicate that, in Russian regions, the weakness of the legal system tends to increase the probability of distressed acquisitions, while other socioeconomic risks negatively affect it. It was also revealed that, in the most developed areas, monotown enterprises are more likely to be rescued by acquisition after management failure than other firms; however, this is not always true for the entire federation and other regions. Based on the empirical evidence obtained from this study, we maintain that, in Russia, distressed acquisitions are ceasing to serve a complementary function to the legal system, mainly because of recent improvements in formal business regulation and practice, as well as the sharp increase of investment risks associated with the economic hardships over the past decade.

The remainder of the paper is organized as follows. The next section develops hypotheses to test in this paper. “Data and Empirical Methodology” section describes the data and empirical methodology. “Results” section reports the results. “Conclusions” section summarizes the major findings and concludes the paper.

## Hypothesis Development

In this section, based on the historical developments and present-day situation in Russia, we present our hypotheses about the impact of the weakness of the legal system, socioeconomic investment risks, and the existence of so-called monotowns (one-company town) on the probability of the distressed acquisitions of Russian firms.

In Russia, economic and commercial disputes among business entities, including disputes between creditors and debtors in the event of corporate bankruptcies, are handled in commercial courts known as arbitration courts. Currently, the arbitration courts are structured in four levels: Trial courts are organized along the lines of the Russian federation (regions) as the courts of first instance. At the second and third levels, there are arbitration courts of appeal and courts of cassation appeal, respectively. Finally, the Supreme Court of the Russian Federation is the court of supervisory appeal (www.arbit.ru).

Noncommercial disputes such as criminal cases are handled by general courts, known as courts of general jurisdictions. Businesspeople have evaluated the effectiveness of arbitration courts more positively than that of the general courts (Frye 2017; Titaev 2012; Hendley et al. 2000). Arbitration courts are known to have relatively more financial and administrative independence than general courts (Bocharov and Titaev 2018).

Nonetheless, the problems associated with Russia’s weak legal/judicial system, such as the length of trial processes, high attorney fees, and the corruption of judges, also apply to the arbitration courts (Burger 2004). Informal intervention by politicians exacerbates the situation, as commercial courts have been subject to political influence. (Gustafsson 2013; Lambert-Mogiliansky et al. 2007). These problems have significantly increased the opportunity cost of using the arbitration court (Burger 2004; Burger and Gitau 2010). Despite major reform in 2014, the assessment of Russia’s system of arbitration has not been favorable (Oda 2019).

As mentioned in the Introduction, distressed acquisitions can be an effective means of avoiding dispute resolution in an arbitration court, or at least minimizing court or third-party interventions (Iwasaki et al. 2021). It has been shown that, in East Asia, stronger creditor rights and a better judicial system increase the likelihood of bankruptcy filings in resolving corporate distress in a country (Claessens et al. 2003), and that both strong creditor and shareholder rights increase the use of bankruptcy relative to acquisition as a mechanism for resolving financial distress (Dahiya and Klapper 2007).

Furthermore, it is costly not only to liquidate a company through bankruptcy procedures but also to establish a new company, which comes with both formal and informal institutional barriers in Russia (Aidis and Adachi 2007; Iwasaki et al. 2016). In Russia, various ‘competing’ informal institutions undermine, rather than complement, the functioning of formal institutions in investor relations (Estrin and Prevezer 2011), and the weakness of the institutional environment has exerted a detrimental impact on entrepreneurial activity (Aidis et al. 2008). In addition, creating and maintaining the necessary personal connections with local influential people are indispensable for doing business in Russia (Ledeneva 2013; Yakovlev and Ivanov 2021). This initial investment could prove large and costly.

Therefore, it could be conjectured that investors will opt for a distressed acquisition when the opportunity cost of liquidating a bankrupt company in accordance with the rules and practices under Russia’s legal system exceeds that of a corporate acquisition. In other words, the weaker the functioning of the legal institutions, the costlier firm bankruptcy and the liquidation of assets would be, hence the stronger the incentive for favoring distressed acquisitions. Therefore, we anticipate the following.

### Hypothesis H1

*Legal weakness is positively associated with the probability of distressed acquisitions.*

The acquisition of a bankrupt company is a pure investment activity. As long as this is the case, the likelihood of distressed acquisition, like any other investment activity, will be largely dependent on the predictability of future cost recovery. That is, the probability of a distressed acquisition can be greatly affected by the investment risks in an overall business environment.

In Russia, there are various socioeconomic risks that obscure the predictability of corporate investment. Risks associated with the country’s economic, financial, political, and social conditions create uncertainties that can be as detrimental as the weak legal system in terms of their impact on the investment climate. To begin with, the intensity of economic fluctuations peculiar to emerging markets casts a shadow on the outlook for regional economic development. The underdevelopment of local financial institutions impairs the certainty of financing. Reliance on bank loans to finance investment needs by companies has been low, and the financial system has not been a strong boost to economic growth (Sutela 2009; Kirdina and Vernikov 2013; Mirkin et al. 2013). Factors such as organized crime, political corruption, and the unreliability of administrative organizations undermine growth and enhance risks (Varese 2001; Volkov 2002; Holmes 2008; Kosals and Maksimova 2015). The protection of property rights, deemed essential for investment, is grossly undermined by widespread cases of corporate raiding, based on illegal and corrupt practices involving business and state actors (Viktorov 2019; Rochlitz 2014; Rochlitz et al. 2020). No doubt, those factors also have a negative impact on investor sentiment (Ledeneva 2006; Pomeranz and Rojansky 2016). As an illustration, according to recent government surveys, around 80% of businesspeople regard doing business in Russia as a risky undertaking: They fear arbitrary criminal investigations and worry about the predatory nature of the state against private business, making them cautious about investing in business expansion (Dumes 2019; Moscow Times 2021; Alekhina 2021; Kornia 2020).

The higher the investment risks related to economics, finance, crime, politics, and administration, as described above, the less the future potential of a business plan to reconstruct bankrupt companies by daringly acquiring distressed firms. In such an environment, the liquidation of financially distressed firms would make more sense than the distressed acquisition of those firms. Therefore, contrary to the impact of the weakness of the legal/judicial system on the probability of distressed acquisition hypothesized above, these socioeconomic risks would induce investors-stakeholders to decide to liquidate rather than put up a company for distressed acquisitions. From the forgoing discussion, we present the following hypothesis.

### Hypothesis H2

*Socioeconomic investment risks are negatively associated with the probability of distressed acquisitions.*

In order to fully grasp the Russian economy, one of the inevitable issues to consider is the monotowns located throughout the country. Monotowns are urban settlements established around a single industry or a core company. They emerged more intentionally, rather than spontaneously, as a result of the Soviet government's industrial allocation policy during the socialist era. The rationale was the policy of economic development of isolated but resource-rich locations and spatial division of labor, with strategic and political logic during the Soviet period (World Bank 2010; Uskova 2012). Following the fall of the Soviet Union, Russia’s monotowns, which have their own difficulties, remain key to the Russian economy (Knox 2016; Zubarevich 2011; Commander 2018). As will be described below, the Russian Federation government issued an order in 2014 to designate more than 300 municipalities as monotowns.^3^ Around 13.5 million people—about 9.2% of Russia’s population—live in those monotowns. Many of Russia’s large companies are major employers in monotowns. They include the world’s leading nickel producer, Norilisk Nickel; Russia’s leading metal producers, such as Severstal, Novlipetsk Metal, and Mechel; the largest coal company, SUEK; as well as major producers in the automotive industry, Avtovaz and Kamaz. The list goes on (Nesterov 2019; Voluiskaia 2019).

A typical Russian monotown is located in a remote area and has a single core company with a high concentration of employers that basically are responsible for the local services supporting the lives of workers and their families. A corporate bankruptcy in such a monotown can have a tremendous adverse effect on the lives of citizens. Therefore, it has been pointed out that monotowns are more likely than other areas to receive political protection and policy support (World Bank 2010; Crowley 2016; Nesterov 2019; TASS 2016). A perceived significance of tackling economic problems in monotowns is reflected in the establishment of the Monotown Development Fund in 2014, founded by the state development corporation Vnesheconombank (VEB), with a view to facilitating necessary conditions to create new jobs and attracting investments in the monotowns.^4^

Given the possible social disruption and shocks that failure and closure of the business would cause, it could be conjectured that not only the bankruptcy of a company located in a monotown is less likely to occur, but even if it did, it is also likely that the company would be bailed out by acquisition in order to minimize the detrimental impact on the entire socioeconomic wellbeing of the monotown. Therefore, we make the following prediction.

### Hypothesis H3

*The probability of distressed acquisitions of firms located in monotowns is higher than that in other places.*

Russia, the world’s largest country, is composed of over 80 constituent subjects of the federation. There are regional differences in socioeconomic conditions; therefore, the investment climate, including the legal system, is quite diverse. In what follows, we will conduct statistical and quantitative analyses to empirically test our three hypotheses.

## Data and Empirical Methodology

To empirically test the three hypotheses proposed in the previous section, we utilize a large dataset of Russian firms. The dataset contains firm-level variables extracted from the Orbis database of Bureau van Dijk (BvD)^5^ and region-level variables constructed that refer to information on regional investment conditions provided by the rating agency Expert RA and a decree of the Federal Government concerning monotowns in Russia.

In the Orbis database, we identified a total of 93,260 Russian business firms that satisfy the next three conditions: (a) they were operating at the end of 2006, (b) their survival status is traceable until the end of 2019, and (c) their location is identifiable at the city/town level. In respect to survival status, we categorized each entry firm as either (A) a company that maintained operations through the observed period without financial distress (i.e., survivors), (B) a company that was “bankrupted,” “liquidated,” or “dissolved” without any subsequent legal status change before the end of the observed period, (C) a company that became “dormant” during the observed period, or (D) a company that became “dormant,” “bankrupt,” “liquidated,” or “dissolved” with a subsequent legal status change to “merged/taken over” within the observed period. We classified firms that fall into category D as distressed acquisitions.^6^

Concerning the location of the companies, the sample firms are registered in 882 cities/towns of 81 federal constituent entities (i.e., republics, territories, regions, autonomous areas, or federal cities). We confirmed that their distribution by location at the level of federal constituent entities is almost consistent with the official statistics of the numbers of firms and organizations at the end of 2006 (Rosstat 2007), except for a somewhat higher percentage of firms in Moscow (35.1% in the sample as opposed to 23.1% in the official statistics).

**Fig. 2:**
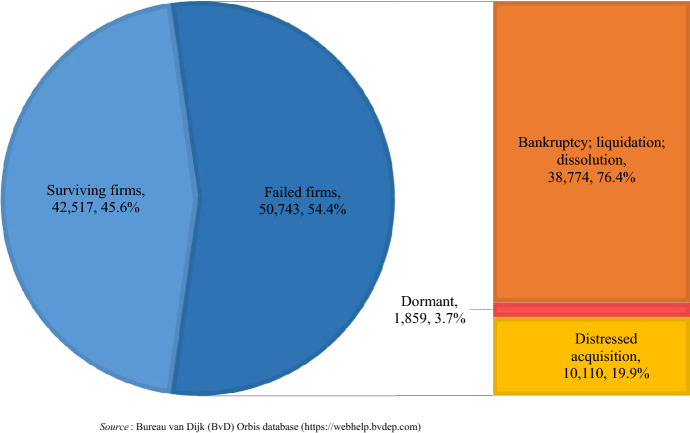
Survival status of 93,260 Russian firms at the end of 2019

**Fig. 3:**
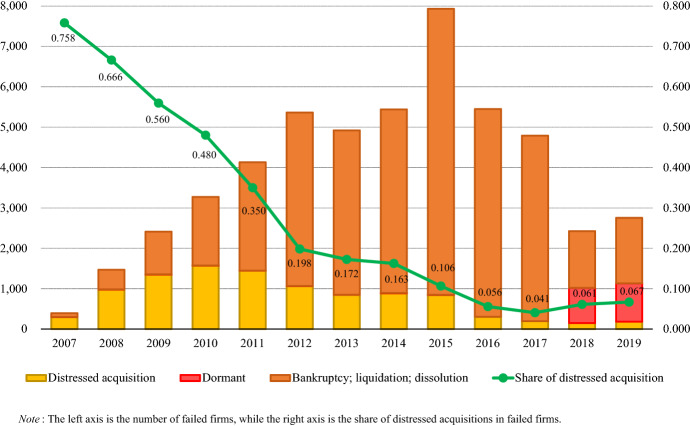
Dynamics of firm failure and distressed acquisitions in Russia during the period from 2007 to 2019

Figure 2 shows the survival status of sample firms at the end of 2019. Of 93,230 firms, 50,743 or 54.4% failed during the 13 years starting in 2007. Additionally, 38,774, or 76.4% of distressed companies, disappeared following legal proceedings; and 1859, or 3.7% of failed firms, were found to be dormant. The remaining 10,110, or 19.9%, were rescued by acquisition. According to Fig. 3, firm failure began to increase markedly in 2008, the year of the global financial crisis, and peaked in 2015, one year after Russia’s annexation of Crimea in 2014. In the following two years, the number of failed firms remained high, finally settling below 3000 in 2018–19. It is noteworthy that, during the observation period, the share of distressed acquisitions in failed firms showed a marked downward trend from 75.8% in 2007 to 6.7% in 2019. In other words, distressed acquisitions have lost much of their role as a means of dealing with the management failure of Russian companies over the past decade.

**Fig. 4:**
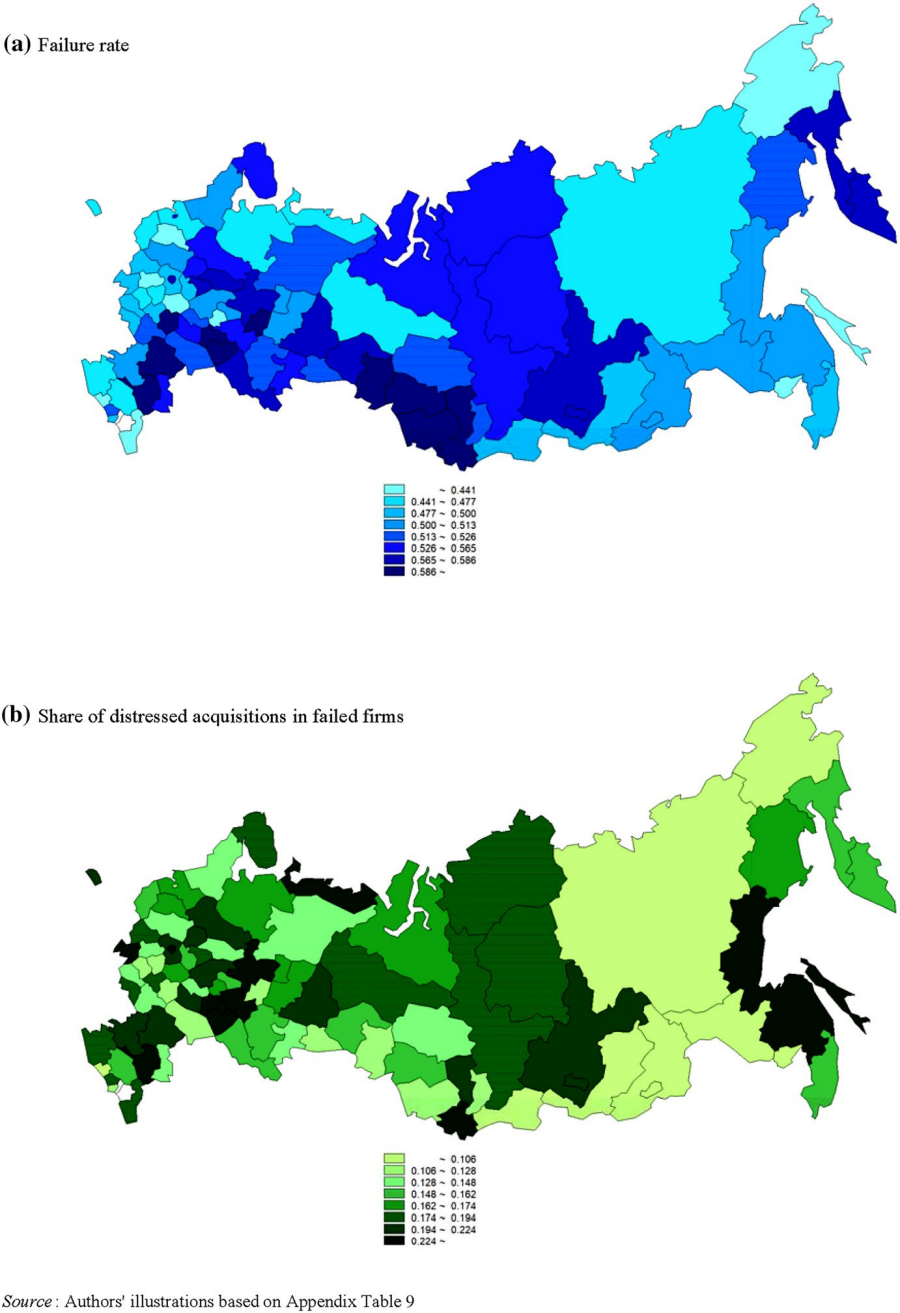
Regional distribution of failure rate and share of distressed acquisitions in failed firms during the period of 2007–2019

Table 1 exhibits the survival status of 93,260 companies and share of distressed acquisitions in failed firms by sector and federal district.^7^ Among five industry sectors, the share of distressed acquisitions is highest in financial services (22.8%), followed by nonfinancial services (22.0%). The other three sectors show a ratio lower by about 5–6% than that of the former two industries. Of eight federal districts, the share of distressed acquisitions in the Central Federal District is the highest, at 22.3%, followed by 20.2% in the Volga Federal District and 19.9% in the Southern Federal District. The ratios in the other five districts range between 14.2 and 16.9%. At the same time, Table 2 and Fig. 4 demonstrate that both the failure rate and share of distressed acquisitions greatly vary within each federal district, suggesting that the factors at the level of federal constituent entities may significantly influence the destiny of Russian companies, as we argued in the previous section.

**Table 1:** Survival status of 93,260 firms and share of distressed acquisitions in failed firms in Russia by sector and federal district, 2007–2019 (*Source*: Bureau van Dijk (BvD) Orbis database (https://webhelp.bvdep.com))

	Number of firms operating at the end of 2006 (*N*)	Number of surviving firms (survivors) by the end of 2019 (*A*)	Number of failed firms by the end of 2019				Failure rate (*F*/*N*)	Share of distressed acquisitions in failed firms (*D*/*F*)
			Total failed firms (*F*=*B*+*C*+*D*)	Bankruptcy, liquidation, dissolution (*B*)	Dormant (*C*)	Distressed acquisition (*D*)		
All firms	93,260	42,517	50,743	38,774	1859	10,110	0.544	0.199
*Breakdown by sector (NACE Rev. 2 section)*								
Agriculture, forestry, and fishing (section A)	6114	3414	2700	2192	48	460	0.442	0.170
Mining, energy, and manufacturing (sections B–E)	23,176	12,626	10,550	8397	308	1845	0.455	0.175
Construction (section F)	13,144	5054	8090	6445	297	1348	0.615	0.167
Nonfinancial services (sections G–J, L–S)	50,451	21,280	29,171	21,568	1199	6404	0.578	0.220
Financial services (section K)	375	143	232	172	7	53	0.619	0.228
*Breakdown by federal district*								
Central Federal District	46,485	20,670	25,815	18,968	1082	5765	0.555	0.223
Northwestern Federal District	9493	4603	4890	4048	149	693	0.515	0.142
Southern Federal District	5614	2764	2850	2168	114	568	0.508	0.199
North Caucasus Federal District	1303	716	587	486	15	86	0.450	0.147
Volga Federal District	12,678	5892	6786	5224	188	1374	0.535	0.202
Ural Federal District	6394	2948	3446	2768	97	581	0.539	0.169
Siberian Federal District	8035	3272	4763	3832	145	786	0.593	0.165
Far East Federal District	3258	1652	1606	1280	69	257	0.493	0.160

**Table 2:** Failure rate and share of distressed acquisitions in failed firms in Russia by federal district, 2007–2019 (*Source*: Authors’ computations based on Appendix Table 9)

	Number of regions	Failure rate						Share of distressed acquisitions in failed firms					
		Mean	S.D.	Median^a^ (Region)	Max.^b^ (Region)	Min.^b^ (Region)	Coefficient of variation	Mean	S.D.	Median^a^ (Region)	Max.^b^ (Region)	Min.^b^ (Region)	Coefficient of variation
All regions	81	0.514	0.072	0.512 (Khabarovsk Territory, Republic of Karelia)	0.803 (Republic of Altai)	0.341 (Republic of Daghestan)	0.141	0.163	0.062	0.161 (Republic of Mari El)	0.333 (Nenets Autonomous Area)	0.000 (Chukotka Autonomous Area, Jewish Autonomous Region, Republic of Tuva)	0.379
Central Federal District	18	0.505	0.054	0.490 (Lipetsk Region, Smolensk Region)	0.601 (Tambov Region)	0.412 (Kaluga Region)	0.107	0.166	0.040	0.160 (Ryazan Region, Vladimir Region)	0.243 (Bryansk Region)	0.112 (Tambov Region)	0.241
Northwestern Federal District	11	0.483	0.048	0.462 (Arkhangelsk Region)	0.543 (Vologda Region)	0.409 (Novgorod Region)	0.100	0.179	0.058	0.167 (Arkhangelsk Region, Novgorod Region)	0.333 (Nenets Autonomous Area)	0.118 (St. Petersburg Federal City)	0.326
Southern Federal District	6	0.543	0.096	0.533 (Rostov Region)	0.676 (Republic of Kalmykia)	0.432 (Republic of Adygeya)	0.177	0.192	0.072	0.198 (Krasnodar Territory, Rostov Region)	0.304 (Republic of Kalmykia)	0.094 (Republic of Adygeya)	0.378
North Caucasus Federal District	5	0.446	0.066	0.459 (Stavropol Territory)	0.517 (Kabardino–Balkarian Republic)	0.341 (Republic of Daghestan)	0.149	0.143	0.033	0.149 (Stavropol Territory)	0.174 (Kabardino–Balkarian Republic)	0.098 (Karachayevo–Circassian Republic)	0.233
Volga Federal District	14	0.532	0.053	0.525 (Penza Region, Republic of Mordovia)	0.615 (Republic of Udmurtia)	0.401 (Chuvash Republic)	0.100	0.189	0.058	0.164 (Chuvash Republic, Perm Territory)	0.281 (Ulyanovsk Region)	0.118 (Republic of Udmurtia)	0.307
Ural Federal District	6	0.531	0.042	0.537 (Chelyabinsk Region, Yamal–Nenets Autonomous Area)	0.569 (Tyumen Region)	0.452 (Khanty–Mansi Autonomous Area–Yugra)	0.080	0.158	0.032	0.158 (Tyumen Region, Yamal–Nenets Autonomous Area)	0.196 (Sverdlovsk Region)	0.113 (Kurgan Region)	0.203
Siberian Federal District	10	0.590	0.092	0.579 (Altai Territory, Irkutsk Region)	0.803 (Republic of Altai)	0.478 (Republic of Tuva)	0.156	0.148	0.072	0.143 (Novosibirsk Region, Tomsk Region)	0.254 (Republic of Altai)	0.000 (Republic of Tuva)	0.485
Far East Federal District	11	0.474	0.063	0.494 (Primorsky Territory)	0.567 (Kamchatka Territory)	0.346 (Jewish Autonomous Region)	0.133	0.116	0.083	0.097 (Zabaikalsk Territory)	0.254 (Sakhalin Region)	0.000 (Chukotka Autonomous Area, Jewish Autonomous Region)	0.720

Data are not available for the Republic of Ingushetia and the Chechen Republic due to the lack of firm-level observations

^a^If two regions are mentioned in parentheses, it denotes that these regions share a median value or the median value is computed using their rates

^b^If two or more regions are mentioned in parentheses, it denotes that these regions share the same rate

As key variables for testing the hypotheses, we constructed eight region-level variables. The first six variables originated in the Expert RA rating of investment risks in Russian regions from the perspective of the legal system, economy, finance, crime, politics, and administration, in which federal constituent entities are ordered from 1 (best) to 83 (worst).^8^ The regional rating of the legal system is used to test Hypothesis H1, while the other five ratings examine Hypothesis H2. In order to estimate the overall effect of the socioeconomic risk on distressed acquisitions, we also employ the first principal component score of the five ratings from economy to administration as a comprehensive index of the socioeconomic risk in Russian regions.^9^

To test Hypothesis H3, we use a dummy variable for firms located in a monotown as the eighth region-level variable. *Monotowns* are defined as single-industry municipalities designated in the government decree of July 29, 2014, which lists a total of 334 cities/towns subject to the special attention of the federal government from the viewpoint of regional development policy. The variable gives a value of 1 to firms located in one of these 334 municipalities. We found that, of 93,260 sample firms, 5383, or 5.8%, are registered in these monotowns.

To estimate the eight region-level variables, we follow the empirical methodology adopted in Iwasaki et al. (2021). Specifically, we estimate a model that regards the decision to acquire a distressed firm to be the result of a dichotomous choice: to rescue a distressed firm by acquisition, or not to. The literature argues that this dichotomization may cause a heterogeneity bias problem. In addition, the decision to acquire a distressed firm gives rise to a self-selection problem (Van de Ven and Van Praag 1981). Our model deals with these two econometric issues by employing the Heckman two-step procedure, which allows us to estimate equations of the selection model and the outcome model simultaneously. More concretely, we estimate the next set of equations:

1 $$ {\text{Distress}}\;{\text{model}}{:}\;\Pr \left( {D_{i} = 1|Z_{ij} } \right) = \mu + \alpha Z_{ij} + \varepsilon_{i} , $$

2 $$ {\text{Acquisition}}\;{\text{model}}{:}\;\Pr \left( {A_{i} = 1|W_{ij} } \right) = \eta + \beta W_{ij} + \lambda_{i} + \epsilon_{i} , $$

where in Eq. (1), *D*_*i*_ is the dichotomous variable that assigns a value of 1 to firms distressed during the observation period of 2007–2019, and *Z*_*ij*_ is a set of variables that affect the probability of financial distress of the *i*-th firm in the *j*-th region. Meanwhile, in Eq. (2), *A*_*i*_ is the dichotomous variable, which equals 1 if a distressed firm is acquired and 0 otherwise, for each *i*-th distressed firm; *W*_*ij*_ is a set of variables that influence the decision to acquire the *i*-th firm; factor *λ*_*i*_ is obtained from the first-stage estimation and controls for sample selection bias; *μ* and *η* are constant terms; and *ε*_*i*_ and $\epsilon_{i}$ represent error terms that satisfy the following condition:

3 $$ \left( {\begin{array}{*{20}c} {\varepsilon_{i} } \\ {\epsilon_{i} } \\ \end{array} } \right) \sim i.i.d.\left( {\left( {\begin{array}{*{20}c} 0 \\ 0 \\ \end{array} } \right),\left( {\begin{array}{*{20}c} {\sigma_{\varepsilon }^{2} } & {\rho_{\epsilon \varepsilon } } \\ {\rho_{\varepsilon \epsilon } } & {\sigma_{\epsilon }^{2} } \\ \end{array} } \right)} \right). $$

In order to obtain unbiased estimates of the region-level variables, both Eqs. (1) and (2) include on the right-hand side a rich set of variables that capture firm-level characteristics and industry fixed effects. Firm-level control variables are selected in accordance with the estimation results in Iwasaki and Kim (2020) and Iwasaki et al. (2021). To be specific, both distress and acquisition models control for the legal form of incorporation, ownership structure, financial performance, listing on the stock market, fund-raising capacity, firm size/age, and business network/diversification. The distress model additionally controls for managerial discretion and the corporate governance system to take account of the capability of managers, board directors, and auditors to avoid financial distress of their company. Industry fixed effects are also controlled for at the NACE division level.

Consistent with Iwasaki and Kim (2020) and Iwasaki et al. (2021), all region-level variables and firm-level control variables take a value in 2006 to assess the predictive power of the initial conditions that is the empirical focus in this study. This approach enables us to avoid or significantly mitigate the issue of potential endogeneity. Table 3 lists the name, definition, and descriptive statistics of the independent variables.

**Table 3:** Definitions and descriptive statistics of independent variables used in the empirical analysis (*Source*: Authors’ compilation and estimation. Region-level data—from legal weakness to administrative risk—was obtained from the website of the rating agency Expert RA (http://www.raexpert.ru/ratings/). Firms located in company towns are specified by the authors in reference to Government Decree No. 1398-r of July 29, 2014 “On the list of single-industry municipalities of the Russian Federation (monotowns)” (Pacпopяжeниe oт 29 июля 2014 гoдa И 1398-p «O пepeчнe мoнoпpoфильныx мyниципaльныx oбpaзoвaний Poccийcкoй Фeдepaции (мoнoгopoдoв)»). Firm-level raw data were extracted from the Bureau van Dijk (BvD) Orbis database. For details of the database and data, see the BvD website: https://webhelp.bvdep.com. Alternative region-level statistics were obtained from the website of the Federal State Statistic Service of the Russian Federation (http://www.gks.ru/))

Variable name	Definition	Descriptive statistics^a^		
		Mean	S.D.	Median
*Region-level variables*				
Legal weakness	Expert RA region rating of investment risk in the legal system	52.476	28.347	58
Economic risk	Expert RA region rating of investment risk in the economy	22.140	23.467	14
Financial risk	Expert RA region rating of investment risk in finance	19.556	22.284	7
Criminal risk	Expert RA region rating of investment risk in crime	35.313	22.833	32
Political risk	Expert RA region rating of investment risk in politics	48.608	19.343	58
Administrative risk	Expert RA region rating of investment risk in administration	26.363	22.157	17
Comprehensive socioeconomic risk	First principal component score of the variables from economic risk to administrative risk^b^	0.004	1.553	− 0.295
Location in a monotown	Dummy for firms located in a mono-town	0.058	0.233	0
*Firm-level control variables*				
Open joint-stock company	Dummy variable for open (public) joint-stock companies (OAO)	0.104	0.306	0
Closed joint-stock company	Dummy variable for closed (private) joint-stock companies (ZAO)	0.142	0.350	0
Limited liability company	Dummy variable for limited liability companies (OOO)	0.678	0.467	1
Large shareholding	Dummy for firms with a dominant and/or block shareholder(s)	0.905	0.293	1
Foreign ownership	Dummy for firms with foreign investors as the ultimate owner^c^	0.009	0.095	0
Federal state ownership	Dummy for firms with the Russian federal government as the ultimate owner^c^	0.019	0.137	0
Regional state ownership	Dummy for firms with a Russian regional government as the ultimate owner^c^	0.032	0.175	0
Managerial discretion	BvD independent indicator (0: D; 1: C; 2: C+; 3: B−; 4: B; 5: B+; 6: A−; 7: A; 8: A+)^d^	3.440	3.646	0
Board size	Number of recorded members of the board of directors	1.491	1.856	1
International audit firm	Dummy for firms that employ an international audit firm as an external auditor	0.001	0.026	0
Large Russian audit firm	Dummy for firms that employ a large Russian audit firm as an external auditor	0.001	0.032	0
Local Russian audit firm	Dummy for firms that employ a local Russian audit firm/auditor as an external auditor	0.007	0.082	0
ROA	Return on total assets (%)^e^	10.475	20.250	5.960
Gross margin	Gross margin (%)^f^	13.465	18.617	9.740
Listing on the stock market	Dummy variable for listed companies	0.007	0.081	0
Gearing	Gearing (%)^g^	74.962	163.246	2.000
Firm size	Natual logarithm of total assets	10.070	1.681	10.028
Firm age	Natual logarithm of years in operation	1.873	0.783	1.946
Business network	Number of subsidiaries	0.661	3.172	0
Business diversification	Number of operating industries according to the NACE Rev 2 secondary codes	6.804	3.763	7
*Alternative region-level variables*^h^				
Economic growth	Growth rate of gross regional product (%)	8.242	2.312	8.770
Firm population density	Natual logarithm of the number of firms per one million residents	8.449	0.746	8.044
Access to finance	Natual logarithm of the number of financial organizations per one million residents	2.975	0.342	3.007
Financial soundness of the corporate sector	Proportion of profitable companies to total firms (%)	70.199	5.618	71.100
Government size	Natual logarithm of civil servants per one million residents	6.868	0.338	6.812
Judicial sector size	Natual logarithm of the number of staff members in judicial and prosecutorial institutions per one million residents	4.717	0.380	4.745

The independent variables capture the region-wide and firm-level initial conditions in 2006 for firm failures and distressed acquisitions observed during the period of 2007–2019. The correlation matrix of the variables is reported in Appendix Table 11

^a^Computed using firm-level data

^b^Appendix Table 10 reports the estimation results of the principal component analysis

^c^In the ORBIS database, *ultimate owner* is defined as “the individual or entity that owns more than 50.01% of the equity directly or via subsidiaries”

^d^Class A: Definition—Attached to any company with known recorded shareholders, none of which have more than 25% of direct or total ownership [A+: Companies with 6 or more identified shareholders (of any type) whose ownership percentage is known; A: Same as above, but includes companies with 4 or 5 identified shareholders; A−: Same as above, but includes companies with 1 to 3 identified shareholders]. Class B: Definition—Attached to any company with a known recorded shareholder, none of which has an ownership percentage (direct, total, or calculated total) over 50%, but which has one or more shareholders with an ownership percentage above 25%. The further qualifications of B+, B, and B− are assigned according to the same criteria relating to the number of recorded shareholders as for indicator A. Class C: Definition—Attached to any company with a recorded shareholder with total or a calculated total ownership over 50%. The qualification C+ is attributed to C companies in which the summation of direct ownership percentage (all categories of shareholders included) is 50.01% or higher. Indeed, this means that the company surely does not qualify under Independent Indicator D (since it cannot have an unknown direct shareholder with 50.01% or higher). Class D: Definition—This is allocated to any company with a recorded shareholder with direct ownership of over 50% (quotation from the BvD Orbis database website manual)

^e^Computed using the following formula: (profit before tax/total assets) × 100

^f^Computed using the following formula: (gross profit/operating revenue) × 100

^g^Computed using the following formula: ((non current liabilities + loans)/shareholders’ funds) × 100

^h^These variables are used for supplement regression estimation in Appendix Table 15. The economic growth variable takes the 3-year average of 2004–2006, while others take the value in 2006

As Eq. (3) indicates, the Heckman two-step model assumes that the error terms of Eqs. (1) and (2) are normally distributed with zero mean and variance *δ*^2^ and are correlated with each other. We test the null hypothesis that *ρ* = 0 by a likelihood-ratio test, which compares the log likelihood of the full model with the sum of the log likelihoods for the selection and outcome models. Rejection of the null hypothesis means that the estimators are not biased by a self-selection problem (Annunziata et al. 2019). In the estimation results, we report the Chi-squared statistic of the LR test of independence of equations in addition to the result of a Wald test of the null hypothesis that all coefficients are zero.

## Results

Table 4 shows a univariate comparison between sample firms that fall into the category of bankruptcy/liquidation/dissolution and those in the category of distressed acquisition using the variables introduced in the estimation of the acquisition model.^10^ From this table, we confirm that there exists a statistically significant difference between the two categories of distressed companies in 21 of 23 variables. The test results of the variables from legal weakness to comprehensive socioeconomic risk are consistent with Hypotheses H1 and H2, while that of the variable of location in a monotown does not support Hypothesis H3.

**Table 4:** Univariate comparison of distressed companies with different survival statuses

Variable name	Survival status at the end of 2019				Univariate comparison	
	Bankruptcy, liquidation, dissolution		Distressed acquisition		Test for equality of means (*t*) or test for equality of proportions (*z*)	Wilcoxon rank-sum test (*z*)
	Mean	Median	Mean	Median		
*Region-level variables*						
Legal weakness	52.588	58	57.848	70	− 16.666***	− 17.703***
Economic risk	22.171	13	18.653	6	13.466***	16.278***
Financial risk	19.746	7	16.847	4	11.700***	15.681***
Criminal risk	35.666	33	32.458	17	12.524***	14.534***
Political risk	48.964	58	49.602	58	− 3.021***	− 3.790***
Administrative risk	26.881	17	24.786	17	8.476***	5.117***
Comprehensive socioeconomic risk	0.028	− 0.295	− 0.229	− 1.022	14.677***	16.833***
Location in a monotown	0.060	0	0.048	0	4.446***	4.446***
*Firm-level control variables*						
Open joint-stock company	0.083	0	0.047	0	12.365***	12.365***
Closed joint-stock company	0.125	0	0.115	0	2.597***	2.597***
Limited liability company	0.736	1	0.769	1	− 6.870***	− 6.870***
Large shareholding	0.797	1	0.964	1	− 39.891***	− 39.891***
Foreign ownership	0.005	0	0.014	0	− 9.858***	− 9.858***
Federal state ownership	0.011	0	0.020	0	− 6.991***	− 6.991***
Regional state ownership	0.019	0	0.041	0	− 12.908***	− 12.908***
ROA	7.150	4	9.250	5	− 9.378***	− 11.063***
Gross margin	10.997	7.160	13.187	8.210	− 10.304***	− 9.737***
Listing on the stock market	0.003	0.000	0.002	0.000	1.171	1.171
Gearing	90.004	1.120	71.035	0.040	8.211***	10.742***
Firm size	9.897	9.905	10.051	9.999	− 8.154***	− 6.428***
Firm age	1.696	2	1.577	2	13.533***	13.715***
Business network	0.372	0	0.341	0	1.201	− 0.313
Business diversification	6.800	7	7.155	8	− 8.569***	− 8.960***

*** denotes statistical significance at the 1% level. Table 3 provides definitions and descriptive statistics of variables

Moreover, the test results of firm-level control variables suggest that, as compared with bankrupted, liquidated, or dissolved firms, companies bailed out by acquisition after financial distress tend to be less likely to adopt a joint-stock company but more frequently a limited-liability company as their legal form of incorporation. They are also more likely to include more large shareholders, foreign investors, and the state in their ownership; to have better records in firm performance and fund-raising capabilities; to have larger assets and be younger in the years of operation; and to be more diversified.

In this section, we examine whether the above results are replicable even when these 23 variables are estimated simultaneously in the multivariate regression setting described in the previous section.

### Baseline Estimation

The Heckman second-stage probit estimation results of the acquisition model using a total of 61,016 observations with all necessary independent variables are reported in Table 5. The first-stage estimation results of the distress model are shown in Appendix Table 13.^11^ As shown in the latter table, the distress model is estimated with the variable of location in a monotown in addition to a set of firm-level variables and industry fixed effects, taking into consideration the possibility that monotown enterprises may have a lower risk of financial distress than other firms due to subsidies and/or other protective measures of the government. In Table 5, the LR test of independence of equations rejects the null hypothesis that *ρ* = 0 at a 1% significance level in all seven models, thus, supporting the approach of employing the Heckman two-step procedure to estimate Eqs. (1) and (2).

**Table 5:** Determinants of distressed acquisition: baseline estimation

Model	[1]	[2]	[3]	[4]	[5]	[6]	[7]
*Region-level variables*							
Legal weakness	0.00198***						
	(0.0002)						
Economic risk		− 0.00171***					
		(0.0002)					
Financial risk			− 0.00115***				
			(0.0002)				
Criminal risk				− 0.00145***			
				(0.0002)			
Political risk					− 0.00005		
					(0.0003)		
Administrative risk						− 0.00096***	
						(0.0002)	
Comprehensive socioeconomic risk							− 0.02514***
							(0.0036)
Location in a monotown	0.01829	0.03243	0.02917	0.02555	0.01278	0.01606	0.03385
	(0.0274)	(0.0271)	(0.0271)	(0.0273)	(0.0271)	(0.0269)	(0.0270)
*Firm-level control variables*							
Open joint-stock company	− 0.24660***	− 0.23553***	− 0.23324***	− 0.24092***	− 0.24159***	− 0.23686***	− 0.23109***
	(0.0424)	(0.0408)	(0.0407)	(0.0416)	(0.0415)	(0.0410)	(0.0405)
Closed joint-stock company	− 0.16513***	− 0.16556***	− 0.16420***	− 0.16245***	− 0.16382***	− 0.16516***	− 0.16453***
	(0.0372)	(0.0364)	(0.0363)	(0.0367)	(0.0366)	(0.0363)	(0.0362)
Limited liability company	− 0.13906***	− 0.14255***	− 0.14178***	− 0.14048***	− 0.14450***	− 0.14475***	− 0.14109***
	(0.0344)	(0.0337)	(0.0336)	(0.0341)	(0.0339)	(0.0337)	(0.0336)
Large shareholding	1.91185***	1.91723***	1.92048***	1.91587***	1.92310***	1.92302***	1.91852***
	(0.0703)	(0.0685)	(0.0684)	(0.0694)	(0.0689)	(0.0685)	(0.0683)
Foreign ownership	0.25009***	0.24011***	0.23990***	0.24344***	0.24294***	0.24092***	0.23867***
	(0.0654)	(0.0641)	(0.0640)	(0.0647)	(0.0644)	(0.0641)	(0.0639)
Federal state ownership	0.00924	− 0.00312	− 0.00918	− 0.00511	− 0.01269	− 0.01351	− 0.00674
	(0.0492)	(0.0476)	(0.0473)	(0.0482)	(0.0477)	(0.0474)	(0.0473)
Regional state ownership	0.17223***	0.16011***	0.15293***	0.15949***	0.15280***	0.15137***	0.15514***
	(0.0469)	(0.0448)	(0.0445)	(0.0456)	(0.0452)	(0.0447)	(0.0444)
ROA	0.00571***	0.00577***	0.00573***	0.00573***	0.00570***	0.00572***	0.00577***
	(0.0004)	(0.0004)	(0.0004)	(0.0004)	(0.0004)	(0.0004)	(0.0004)
Gross margin	0.00280***	0.00284***	0.00283***	0.00286***	0.00283***	0.00285***	0.00285***
	(0.0004)	(0.0004)	(0.0004)	(0.0004)	(0.0004)	(0.0004)	(0.0004)
Listing on the stock market	− 0.14726	− 0.15046	− 0.15374	− 0.15843	− 0.15228	− 0.15458	− 0.15575
	(0.1013)	(0.0982)	(0.0979)	(0.0999)	(0.0988)	(0.0981)	(0.0978)
Gearing	− 0.00037***	− 0.00037***	− 0.00037***	− 0.00037***	− 0.00038***	− 0.00037***	− 0.00036***
	(0.00004)	(0.00004)	(0.00004)	(0.00004)	(0.00004)	(0.00004)	(0.00004)
Firm size	0.09235***	0.09125***	0.09063***	0.09208***	0.09125***	0.09099***	0.09094***
	(0.0049)	(0.0048)	(0.0048)	(0.0048)	(0.0048)	(0.0048)	(0.0048)
Firm age	0.20327***	0.21038***	0.20995***	0.20474***	0.20562***	0.20791***	0.21128***
	(0.0180)	(0.0163)	(0.0163)	(0.0173)	(0.0171)	(0.0167)	(0.0161)
Business network	− 0.00164	− 0.00065	− 0.00006	− 0.00099	− 0.00073	− 0.00025	− 0.00004
	(0.0044)	(0.0042)	(0.0042)	(0.0043)	(0.0042)	(0.0042)	(0.0041)
Business diversification	0.00269	0.00408**	0.00445**	0.00434**	0.00558***	0.00487***	0.00363**
	(0.0018)	(0.0017)	(0.0018)	(0.0018)	(0.0018)	(0.0018)	(0.0017)
NACE division-level fixed effects	Yes	Yes	Yes	Yes	Yes	Yes	Yes
*N*	61,016	61,016	61,016	61,016	61,016	61,016	61,016
Censored observations	27,033	27,033	27,033	27,033	27,033	27,033	27,033
Uncensored observations	33,983	33,983	33,983	33,983	33,983	33,983	33,983
Log likelihood	− 51590.930	− 51618.700	− 51637.340	− 51627.970	− 51649.790	− 51640.540	− 51619.930
Wald test (*χ*^2^)	3195.210***	3228.010***	3149.830***	3103.990***	3036.350***	3120.450***	3246.330***
*ρ*	− 0.921	− 0.934	− 0.935	− 0.927	− 0.931	− 0.934	− 0.936
LR test (*χ*^2^)	29.54***	36.98***	37.04***	32.90***	34.95***	35.57***	37.07***

This table contains estimation results of a Heckman probit model with a sample selection of the determinants of distressed acquisition. The coefficient of the constant term is omitted from the table. The estimation results of the first stage are reported in Appendix Table 13. Table 3 provides detailed definitions and descriptive statistics of the independent variables used in the estimation. Figures in parentheses are robust standard errors. The Wald test examines the null hypothesis that all coefficients are zero. The LR test of independence of equations examines the null hypothesis that *ρ* = 0

*** and ** denote statistical significance at the 1% and 5% levels, respectively

**Table 6:** Determinants of distressed acquisition: estimation by industry

Target industry	Agriculture, forestry, and fishing (Section A)		Mining, energy, and manufacturing (Sections B–E)		Construction (Section F)		Nonfinancial services (Sections G–J, L–S)		Financial services (Section K)	
Model	[1]	[2]	[3]	[4]	[5]	[6]	[7]	[8]	[9]	[10]
*Region-level variables*										
Legal weakness	0.00057		0.00098**		0.00222***		0.00206***		0.00101	
	(0.0017)		(0.0005)		(0.0005)		(0.0002)		(0.0058)	
Comprehensive socioeconomic risk		− 0.04486 (0.0361)		− 0.01540* (0.0090)		− 0.01849** (0.0085)		− 0.02803 (0.0040)		− 0.01159 (0.0443)
Location in a monotown	0.07126 (0.2437)	0.16234 (0.2661)	0.01037 (0.0534)	0.02231 (0.0535)	0.04159 (0.0707)	0.04295 (0.0696)	0.00892 (0.0361)	0.02695 (0.0359)	− 0.44675 (0.6380)	− 0.10826 (0.2525)
*Firm-level control variables*										
Open joint-stock company	0.09041 (0.1490)	0.10148 (0.1554)	− 0.08047 (0.1005)	− 0.07320 (0.0987)	− 0.25968** (0.1236)	− 0.23454* (0.1202)	− 0.37066*** (0.0619)	− 0.36128 (0.0608)	− 0.19764 (0.9742)	0.01341 (0.3456)
Closed joint-stock company	0.18554 (0.2106)	0.21321 (0.2208)	0.00699 (0.0960)	0.00864 (0.0951)	− 0.18165 (0.1232)	− 0.16929 (0.1199)	− 0.34792*** (0.0540)	− 0.35270 (0.0534)	0.45080 (0.3661)	0.18510 (0.1703)
Limited liability company	0.23238 (0.1943)	0.26142 (0.2032)	0.08625 (0.0793)	0.08599 (0.0787)	− 0.12659 (0.1167)	− 0.11991 (0.1140)	− 0.35110*** (0.0504)	− 0.35600 (0.0499)		
Large shareholding	2.17445*** (0.3794)	2.13389*** (0.4174)	1.80512*** (0.1583)	1.80382*** (0.1561)	1.73764*** (0.1692)	1.75044*** (0.1669)	1.94858*** (0.1012)	1.94549 (0.1007)	− 0.29545*** (0.4769)	− 0.22029 (0.3806)
Foreign ownership	0.28323	0.30785	0.27130**	0.26736**	0.38907	0.35767	0.19187**	0.18662	0.44530	0.22420
	(0.3726)	(0.3830)	(0.1171)	(0.1160)	(0.3175)	(0.3141)	(0.0852)	(0.0847)	(0.6905)	(0.3234)
Federal state ownership	0.04102 (0.3029)	0.09134 (0.3223)	0.02433 (0.0970)	0.02033 (0.0957)	0.23960* (0.1455)	0.22198 (0.1418)	− 0.03079 (0.0710)	− 0.04003 (0.0703)	− 0.14695 (0.5199)	− 0.18319 (0.5248)
Regional state ownership	− 0.16468 (0.1889)	− 0.12323 (0.2018)	0.18636** (0.0825)	0.17994** (0.0815)	0.27579** (0.1254)	0.27211** (0.1218)	0.06372 (0.0620)	0.05797 (0.0609)		
ROA	0.00615* (0.0032)	0.00591* (0.0034)	0.00524*** (0.0010)	0.00526*** (0.0010)	0.00448*** (0.0009)	0.00450*** (0.0009)	0.00634*** (0.0004)	0.00637 (0.0004)	− 0.00018 (0.0127)	− 0.00018 (0.0051)
Gross margin	0.00795*** (0.0027)	0.00797*** (0.0028)	0.00491*** (0.0010)	0.00487*** (0.0010)	0.00400*** (0.0011)	0.00420*** (0.0011)	0.00093* (0.0005)	0.00098 (0.0005)	− 0.01752* (0.0096)	− 0.00752* (0.0045)
Listing on the stock market	− 0.42780 (0.3207)	− 0.45297 (0.6698)	− 0.02189 (0.1384)	− 0.02478 (0.1371)	− 0.26178 (0.7570)	− 0.24347 (0.6087)	0.64547** (0.3069)	0.60023 (0.3008)	− 0.79932 (1.7000)	0.15346 (0.8918)
Gearing	− 0.00058**	− 0.00056*	− 0.00065***	− 0.00065***	− 0.00033***	− 0.00033***	− 0.00029***	− 0.00028	0.00024	0.00003
	(0.0003)	(0.0003)	(0.0001)	(0.0001)	(0.0001)	(0.0001)	(0.0000)	(0.0000)	(0.0006)	(0.0003)
Firm size	0.13214***	0.12727***	0.12394***	0.12310***	0.04421***	0.04430***	0.09226***	0.09122	0.04283	0.01490
	(0.0321)	(0.0347)	(0.0146)	(0.0145)	(0.0116)	(0.0113)	(0.0059)	(0.0058)	(0.1103)	(0.0450)
Firm age	0.12192	0.10869	0.11172*	0.11344**	0.15537***	0.16570***	0.27317***	0.27576	0.29052	0.13277
	(0.0860)	(0.0932)	(0.0576)	(0.0567)	(0.0382)	(0.0360)	(0.0144)	(0.0138)	(0.2734)	(0.1084)
Business network	− 0.06252*	− 0.06444*	− 0.01579	− 0.01502	0.01686	0.01824	0.00544	0.00627	− 0.04798	− 0.01875
	(0.0377)	(0.0385)	(0.0105)	(0.0103)	(0.0116)	(0.0111)	(0.0055)	(0.0054)	(0.0587)	(0.0230)
Business diversification	0.00319 (0.0078)	0.00279 (0.0081)	− 0.00088 (0.0044)	− 0.00080 (0.0043)	0.00295 (0.0049)	0.00418 (0.0048)	0.00330 (0.0022)	0.00479 (0.0022)	0.02511 (0.0491)	0.00916 (0.0181)
NACE division-level fixed effects	Yes	Yes	Yes	Yes	Yes	Yes	Yes	Yes	Yes	Yes
*N*	4047	4047	15,825	15,825	8616	8616	32,375	32,375	153	153
Censored observations	1277	1277	5417	5417	4636	4636	15,633	15,633	70	70
Uncensored observations	2770	2770	10,408	10,408	3980	3980	16,742	16,742	83	83
Log likelihood	− 2812.211	− 2811.224	− 11974.980	− 11975.770	− 7746.378	− 7756.246	− 28643.650	− 28663.380	− 118.088	− 89.640
Wald test (*χ*^2^)	156.64***	147.33***	694.08***	704.22***	313.07***	293.35***	2204.72***	2180.83	8.76	8.46
*ρ*	− 0.773	− 0.734	− 0.871	− 0.878	− 0.927	− 0.943	− 0.961	− 0.967	− 0.955	− 0.970
LR test (*χ*^2^)	1.55	1.28	8.65***	9.14***	18.81***	20.68***	62.10***	69.86	4.39**	4.42**

This table contains estimation results of a Heckman probit model with a sample selection of the determinants of distressed acquisition. The coefficient of the constant term is omitted from the table. The estimation results of the first stage are reported in Appendix Table 13. Table 3 provides detailed definitions and descriptive statistics of the independent variables used in the estimation. Figures in parentheses are robust standard errors. The Wald test examines the null hypothesis that all coefficients are zero. The LR test of the independence of equations examines the null hypothesis that *ρ* = 0

***, **, and * denote statistical significance at the 1%, 5%, and 10% levels, respectively

**Table 7:** Determinants of distressed acquisition: estimation by region group

Target region group	Central and Northwestern Federal Districts		Southern and North Caucasus Federal Districts		Volga and Ural Federal Districts		Siberian and Far East Federal Districts		Without Moscow Federal City, Moscow Region, St. Petersburg Federal City, and Leningrad Region	
Model	[1]	[2]	[3]	[4]	[5]	[6]	[7]	[8]	[9]	[10]
*Region-level variables*										
Legal weakness	0.00241*** (0.0003)		0.00353* (0.0021)		0.00019 (0.0004)		0.00338*** (0.0013)		0.00060** (0.0003)	
Comprehensive socioeconomic risk		− 0.03479*** (0.0064)		− 0.01881 (0.0233)		− 0.01585 (0.0105)		− 0.15466*** (0.0352)		− 0.02346*** (0.0053)
Location in a monotown	0.09197* (0.0541)	0.12106* (0.0654)	− 0.40989 (0.3182)	− 0.40245 (0.2997)	0.02994 (0.0355)	0.03029 (0.0358)	0.12031 (0.0866)	− 0.13442 (0.1204)	− 0.02321 (0.0265)	− 0.02474 (0.0263)
*Firm-level control variables*										
Open joint-stock company	− 0.24749*** (0.0494)	− 0.23734*** (0.0481)	− 1.13077*** (0.3568)	− 1.12023*** (0.3136)	− 0.08641 (0.0834)	− 0.09131 (0.0846)	− 0.67844*** (0.1832)	− 0.74650*** (0.1833)	− 0.20781*** (0.0577)	− 0.20373*** (0.0566)
Closed joint-stock company	− 0.09443** (0.0434)	− 0.10435** (0.0425)	− 1.01924*** (0.3545)	− 0.99474*** (0.3070)	− 0.20702** (0.0877)	− 0.21653** (0.0895)	− 0.44811** (0.1879)	− 0.53084*** (0.1918)	− 0.17938*** (0.0570)	− 0.17733*** (0.0563)
Limited liability company	− 0.11997*** (0.0414)	− 0.12739*** (0.0404)	− 0.83141*** (0.2961)	− 0.82627*** (0.2688)	− 0.12081 (0.0806)	− 0.12863 (0.0818)	− 0.24691 (0.1613)	− 0.32083* (0.1666)	− 0.10905** (0.0499)	− 0.10968** (0.0496)
Large shareholding	1.84585*** (0.0891)	1.84820*** (0.0875)	1.44292* (0.8738)	1.38246** (0.6423)	2.05614*** (0.1402)	2.04829*** (0.1409)	0.95244*** (0.3185)	1.00766*** (0.3365)	2.03328*** (0.0888)	2.03135*** (0.0883)
Foreign ownership	0.26887*** (0.0759)	0.25952*** (0.0747)	1.66055 (1.0272)	1.59247 (1.0342)	0.17893 (0.1555)	0.18398 (0.1569)	0.28883 (0.3271)	0.27633 (0.3358)	0.25326*** (0.0927)	0.24865*** (0.0921)
Federal state ownership	− 0.03872 (0.0643)	− 0.05903 (0.0624)	0.49735** (0.2539)	0.47384* (0.2456)	− 0.01340 (0.0951)	− 0.00683 (0.0961)	0.30621* (0.1766)	0.35967** (0.1804)	0.00904 (0.0589)	0.01089 (0.0586)
Regional state ownership	0.10589* (0.0612)	0.08962 (0.0590)	0.20284 (0.2222)	0.17856 (0.2133)	0.12586 (0.0789)	0.13266* (0.0797)	0.50721** (0.2087)	0.52725*** (0.2031)	0.19671*** (0.0542)	0.19603*** (0.0535)
ROA	0.00572*** (0.0005)	0.00574*** (0.0005)	0.00040 (0.0039)	0.00021 (0.0030)	0.00607*** (0.0008)	0.00603*** (0.0008)	− 0.00135 (0.0025)	− 0.00103 (0.0025)	0.00550*** (0.0005)	0.00547*** (0.0005)
Gross margin	0.00316*** (0.0005)	0.00321*** (0.0005)	0.00333 (0.0034)	0.00307 (0.0029)	0.00290*** (0.0009)	0.00290*** (0.0009)	0.00208 (0.0016)	0.00204 (0.0017)	0.00258*** (0.0006)	0.00261*** (0.0006)
Listing on the stock market	− 0.33462** (0.1502)	− 0.32074** (0.1445)			− 0.16589 (0.1616)	− 0.16201 (0.1640)	− 0.60122 (1.7422)	− 0.61448 (1.8329)	− 0.05955 (0.1095)	− 0.06805 (0.1089)
Gearing	− 0.00039*** (0.0000)	− 0.00038*** (0.0000)	− 0.00017 (0.0003)	− 0.00016 (0.0002)	− 0.00034*** (0.0001)	− 0.00034*** (0.0001)	− 0.00009 (0.0002)	− 0.00010 (0.0002)	− 0.00042*** (0.0000)	− 0.00041*** (0.0000)
Firm size	0.08948*** (0.0059)	0.08796*** (0.0058)	0.06941 (0.0604)	0.06673 (0.0454)	0.08980*** (0.0103)	0.09029*** (0.0104)	0.08250** (0.0332)	0.09228*** (0.0330)	0.08543*** (0.0070)	0.08514*** (0.0069)
Firm age	0.22283*** (0.0195)	0.22842*** (0.0182)	− 0.21479 (0.1546)	− 0.22505** (0.1023)	0.21457*** (0.0250)	0.20957*** (0.0261)	− 0.29239*** (0.0648)	− 0.28548*** (0.0706)	0.20486*** (0.0201)	0.20441*** (0.0196)
Business network	0.00299 (0.0052)	0.00409 (0.0050)	− 0.06983* (0.0392)	− 0.06757* (0.0383)	− 0.00481 (0.0077)	− 0.00579 (0.0078)	− 0.04232* (0.0220)	− 0.04107* (0.0227)	− 0.00039 (0.0052)	0.00013 (0.0051)
Business diversification	0.00202 (0.0023)	0.00382* (0.0023)	− 0.00034 (0.0095)	0.00030 (0.0093)	0.01035*** (0.0035)	0.00985*** (0.0036)	− 0.00311 (0.0070)	0.00168 (0.0071)	0.00490** (0.0022)	0.00487** (0.0022)
NACE division-level fixed effects	Yes	Yes	Yes	Yes	Yes	Yes	Yes	Yes	Yes	Yes
N	36,784	36,784	4687	4687	12,608	12,608	6937	6937	32,696	32696
Censored observations	16,667	16,667	1846	1846	5434	5434	3086	3086	13,620	13620
Uncensored observations	20,117	20,117	2841	2841	7174	7174	3851	3851	19,076	19076
Log likelihood	− 31182.860	− 31218.810	− 3732.617	− 3734.357	− 10525.980	− 10524.890	− 5750.198	− 5739.482	− 26958.580	− 26949.040
Wald test (*χ*^2^)	2080.62***	2021.01***	269.81***	266.55***	883.45***	870.90***	210.84***	222.98***	1985.54***	2046.00***
*ρ*	− 0.927	− 0.938	0.488	0.554	− 0.983	− 0.978	0.571	0.486	− 0.966	− 0.969
LR test (*χ*^2^)	23.40***	27.61***	0.25	0.32	21.59***	20.78***	1.00	0.81	29.17***	30.52***

This table contains estimation results of a Heckman probit model with a sample selection of the determinants of distressed acquisition. The coefficient of the constant term is omitted from the table. The estimation results of the first stage are reported in Appendix Table 13. Table 3 provides detailed definitions and descriptive statistics of the independent variables used in the estimation. Figures in parentheses are robust standard errors. The Wald test examines the null hypothesis that all coefficients are zero. The LR test of the independence of equations examines the null hypothesis that *ρ* = 0

***, **, and * denote statistical significance at the 1%, 5%, and 10% levels, respectively

**Table 8:** Determinants of distressed acquisition: estimation with focus on firms in monotowns

Model	[1]	[2]	[3]	[4]	[5]	[6]
*Region-level variables*						
Legal weakness	0.00198***		0.00199***		0.00198***	
	(0.0002)		(0.0002)		(0.0002)	
Comprehensive socioeconomic risk		− 0.02518***		− 0.02526***		− 0.02516***
		(0.0036)		(0.0036)		(0.0036)
Location in a monotown	− 0.03708	− 0.01515			0.02237	0.03888
	(0.1759)	(0.1723)			(0.0289)	(0.0284)
Location in a monotown × Firm size	0.00563	0.00499				
	(0.0175)	(0.0172)				
Firms with less than 500 employees in monotowns			0.01870	0.03436		
			(0.0280)	(0.0275)		
Firms with 500–999 employees in monotowns			0.02823	0.04887		
			(0.1789)	(0.1745)		
Firms with 1000–4999 employees in monotowns			− 0.11115	− 0.09418		
			(0.2916)	(0.2835)		
Firms with 5000–9999 employees in monotowns			− 0.02575	− 0.02251		
			(0.5278)	(0.5131)		
Firms with 10000 or more employees in monotowns			0.32429	0.30971		
			(0.5963)	(0.5952)		
Location in a monotown × Federal state ownership					− 0.11880	− 0.11663
					(0.1844)	(0.1810)
Location in a monotown × Regional state ownership					− 0.01972	− 0.03184
					(0.1022)	(0.1004)
*Firm-level control variables*						
Open joint-stock company	− 0.24721***	− 0.23167***	− 0.24916***	− 0.23328***	− 0.24695***	− 0.23136***
	(0.0425)	(0.0405)	(0.0428)	(0.0408)	(0.0425)	(0.0406)
Closed joint-stock company	− 0.16531***	− 0.16471***	− 0.16579***	− 0.16515***	− 0.16528***	− 0.16465***
	(0.0372)	(0.0362)	(0.0373)	(0.0363)	(0.0372)	(0.0362)
Limited liability company	− 0.13909***	− 0.14114***	− 0.13926***	− 0.14132***	− 0.13902***	− 0.14107***
	(0.0344)	(0.0336)	(0.0345)	(0.0337)	(0.0344)	(0.0336)
Large shareholding	1.91161***	1.91832***	1.90964***	1.91681***	1.91156***	1.91841***
	(0.0703)	(0.0683)	(0.0707)	(0.0686)	(0.0704)	(0.0683)
Foreign ownership	0.25036***	0.23896***	0.24940***	0.23815***	0.25030***	0.23881***
	(0.0654)	(0.0639)	(0.0656)	(0.0641)	(0.0654)	(0.0639)
Federal state ownership	0.00933	− 0.00667	0.01089	− 0.00536	0.01583	− 0.00041
	(0.0493)	(0.0473)	(0.0495)	(0.0475)	(0.0502)	(0.0483)
Regional state ownership	0.17275***	0.15560***	0.17487***	0.15733***	0.17440***	0.15856***
	(0.0470)	(0.0444)	(0.0473)	(0.0447)	(0.0480)	(0.0456)
ROA	0.00570***	0.00577***	0.00569***	0.00576***	0.00570***	0.00576***
	(0.0004)	(0.0004)	(0.0004)	(0.0004)	(0.0004)	(0.0004)
Gross margin	0.00280***	0.00285***	0.00280***	0.00285***	0.00280***	0.00285***
	(0.0004)	(0.0004)	(0.0004)	(0.0004)	(0.0004)	(0.0004)
Listing on the stock market	− 0.14666	− 0.15520	− 0.14426	− 0.15272	− 0.14691	− 0.15539
	(0.1014)	(0.0979)	(0.1024)	(0.0988)	(0.1014)	(0.0979)
Gearing	− 0.00037***	− 0.00036***	− 0.00037***	− 0.00036***	− 0.00037***	− 0.00036***
	(0.00004)	(0.00004)	(0.00004)	(0.00004)	(0.00004)	(0.00004)
Firm size	0.09209***	0.09071***	0.09250***	0.09108***	0.09236***	0.09094***
	(0.0050)	(0.0048)	(0.0049)	(0.0048)	(0.0049)	(0.0048)
Firm age	0.20309***	0.21112***	0.20190***	0.21015***	0.20302***	0.21111***
	(0.0181)	(0.0161)	(0.0184)	(0.0164)	(0.0181)	(0.0161)
Business network	− 0.00165	− 0.00005	− 0.00181	− 0.00017	− 0.00168	− 0.00007
	(0.0044)	(0.0041)	(0.0044)	(0.0042)	(0.0044)	(0.0041)
Business diversification	0.00267	0.00362**	0.00272	0.00367**	0.00270	0.00364**
	(0.0018)	(0.0017)	(0.0018)	(0.0017)	(0.0018)	(0.0017)
NACE division-level fixed effects	Yes	Yes	Yes	Yes	Yes	Yes
N	61,016	61,016	61,016	61,016	61,016	61,016
Censored observations	27,033	27,033	27,033	27,033	27,033	27,033
Uncensored observations	33,983	33,983	33,983	33,983	33,983	33,983
Log likelihood	− 51590.750	− 51619.760	− 51589.610	− 51618.680	− 51590.120	− 51619.180
Wald test (*χ*^2^)	3191.770***	3243.350***	3166.700***	3220.530***	3190.320***	3242.640***
*ρ*	− 0.921	− 0.936	− 0.919	− 0.934	− 0.921	− 0.936
LR test (*χ*^2^)	29.63***	37.15***	28.59***	35.97***	29.07***	36.57***

This table contains estimation results of a Heckman probit model with a sample selection of the determinants of distressed acquisition. The coefficient of the constant term is omitted from the table. The estimation results of the first stage are reported in Appendix Table 13. Table 3 provides detailed definitions and descriptive statistics of the independent variables used in the estimation. Figures in parentheses are robust standard errors. The Wald test examines the null hypothesis that all coefficients are zero. The LR test of independence of equations examines the null hypothesis that *ρ* = 0

*** and ** denote statistical significance at the 1% and 5% levels, respectively

In Model [1] of Table 5, the variable of legal weakness is estimated to be statistically significant and positive. This result implies that the weaker the legal system is in a region, the higher the probability of distressed acquisitions in line with Hypothesis H1 and the univariate test result in Table 4. Actually, the coefficient of legal weakness indicates that the likelihood that a distressed firm located in the region with the weakest legal system (ranked 83rd in the Expert rating) is bailed out by a merger with another company is 16.2% higher than in the region with the most reliable legal system (ranked 1st).

In contrast, the investment risk variables—except for the political one—show a significant and negative estimate in Models [2] to [6], suggesting that the probability a distressed firm will be rescued by acquisition is lower in regions with higher investment risks, which is consistent with Hypothesis H2 and the test results in Table 4. The impact of economic risk on distressed acquisitions is the largest, followed by that of criminal risk and financial risk. There is a notable gap in effect size between these three variables and the variable of administrative risk. The comprehensive socioeconomic risk in Model [7] also represents a significantly negative estimate, indicating that overall investment risk tends to strongly restrain the rescue of failed firms by acquisition.

It is noteworthy to point out, in this regard, that the statistical significance of legal weakness is much higher than that of the investment risk variable. In fact, the *t*-value of legal weakness is 8.37, while that of the risk variables ranges between − 0.20 (political risk) and − 6.99 (economic risk). This result suggests that, in Russia, the legal factor is extremely crucial for investors’ decisions to acquire distressed firms or to abandon them.

In all seven models of Table 5, the variable of location in a monotown shows a positive estimate, which is in agreement with Hypothesis H3. Its statistical significance, however, does not reach even the 10% level. Accordingly, we judge that the hypothesis that companies located in monotowns are more likely to be acquired after failure as compared with firms in other places is not empirically supported. In addition, Appendix Table 13 shows that the variable of location in a monotown in the distress model paired with Model [1] of Table 5 is estimated with a negative coefficient as we expect, but, again, it is statistically insignificant. In other words, there is no difference in the frequency of firm failures and distressed acquisitions between single-industry municipalities and other places, if other conditions are held constant.

Estimates of the firm-level control variables provide additional insights into distressed acquisitions in Russia. More concretely, we found that a more open legal form of incorporation promotes the liquidation rather than the acquisition of distressed firms. In fact, according to Model [1] of Table 5, the probabilities of rescuing open joint-stock companies, closed joint-stock companies, and limited liability companies by acquisition after management failure are 24.7%, 16.5%, and 13.9% lower, respectively, than those of other more closed corporate forms (cooperatives, partnerships, etc.). As argued in Iwasaki and Kim (2020), this fact may be closely related to the differences in the transferability of ownership between different legal forms of incorporation.

Moreover, the estimation results in Table 5 indicate that ownership by large shareholders, foreign investors, and regional governments is positively related to the probability of distressed acquisitions, while ownership by the federal government has no impact on it. The asymmetrical attitude between the central and local governments toward failed companies is a fact worth emphasizing, as it is key to understanding the roles of each government in regional industrial policies.

Further, our baseline estimation also revealed that, in Russia, the better the financial performance of a company, the larger its size, the longer it has been in operation, and the more diversified its business, the higher its probability of being acquired after management failure. These findings suggest that potential firm value is quite an important element that determines whether a financially distressed company will continue to exist.^12^

### Estimation by Industry and Region Group

Next, we question whether the findings obtained from the baseline estimation are general across different industrial sectors and regional areas.

Table 6 represents the estimation results by industry. In this table, Models [3] to [8] show a statistically significant estimate of either the variable of legal weakness or comprehensive socioeconomic risk with a sign consistent with our predictions. Hence, it is proved that Hypotheses H1 and H2 well capture the reality of the mining, energy, and manufacturing; construction; and nonfinancial service industries in Russia. In contrast, these two variables are estimated to be insignificant in Models [1], [2], [9], and [10], suggesting that regional factors related to the legal system and other socioeconomic environments do not strongly affect the probability of acquisition of distressed firms in the primary and financial service industries. Further, the variable of location in a monotown is statistically insignificant in all models in Table 6 and in the corresponding distress models in Appendix Table 13 and, accordingly, does not support Hypothesis H3.

The estimation results by region group are reported in Table 7. Here, in Models [1] to [8], eight federal districts are classified into four groups, which take account their similarity and heterogeneity of socioeconomic characteristics, as in Iwasaki and Kumo (2020). From these models, we confirm that Hypotheses H1 and H2 well explain the likelihood of distressed acquisitions in three- and two-region groups, respectively. In other words, legal weakness is less likely to differentiate the probability to bail out failed firms by acquisition “within” the Volga and Ural Federal Districts. The same applies to the comprehensive socioeconomic risk in the case of the Southern and North Caucasus Federal Districts and the case of the Volga and Ural Federal Districts. Hypothesis H3 is supported with significant and positive estimates of the variable of location in a monotown in Models [1] and [2], which implies that, within the bounds of the Central and Northwestern Federal Districts, monotown companies are more likely to be rescued by acquisition after financial distress than their counterparts in other places. The paired distress model in Appendix Table 13 shows that location in a monotown negatively affects the probability of failure of firms in the Central and Northwestern Federal Districts, which is in line with our expectation. These results indicate that monotown enterprises in the most-developed areas enjoy more favorable conditions—including state support—than those in the other areas to keep their existence.

In Table 7, as an additional robustness check, we also tested the extent to which sample firms in metropolitan areas affect the empirical results by excluding Moscow Federal City, Moscow Region, St. Petersburg Federal City, and Leningrad Region from the target regions. Models [9] and [10] show the results. In these two models, both the variables of legal weakness and comprehensive socioeconomic risk display a statistically significant estimate with the predicted sign, while the variable of monotown location is given an insignificant coefficient, suggesting that, in Russia, the logic of distressed acquisition applies commonly to both metropolitan and non-metropolitan firms.^13^

Tables 6 and 7 also demonstrate that the firm-level characteristics that strongly affect the likelihood of distressed acquisitions greatly vary across industries and region groups. We found that large shareholding, financial performance, fund-raising capability, and firm size/age exert a significantly consistent impact in most industries and region groups, while the impacts of legal form of incorporation, state ownership, listing on the stock market, and business network/diversification are limited in specific sectors and region groups. The same observations apply to the estimation results of distress models in Appendix Table 13. In addition to the estimates of region-level variables, these results also provide insights for understanding the sectoral and regional heterogeneity of the Russian economy.

### Estimation with Focus on Firms in Monotowns

Finally, we reexamine our prediction regarding firms in monotowns using a series of extended models. As reported in the previous subsections, the variable of location in a monotown is estimated to be insignificant in every model except for those limited to firms in the Central and Northwestern Federal Districts. We argue that this is presumably due to the heterogeneity among monotown enterprises from the viewpoint of firm size and ownership structure, assuming that, in monotowns, companies with large assets or large numbers of employees or that are owned by the state are less likely to fail and more likely to be bailed out by acquisition—even after failure—as compared with small private firms.

To test the above assumption, we extend both the distress and acquisition models either (a) by adding an interacted variable between location in a monotown and asset size (i.e., the variable of firm size), (b) by replacing the variable of location in a monotown with a set of dummy variables that classify monotown companies into five categories in terms of total number of employees, or (c) by adding interacted variables of location in a monotown with federal state ownership and regional state ownership and estimating these newly introduced variables in the right-hand side of regression equations using all available observations.

The results are shown in Table 8 and the three columns farthest right in Appendix Table 13. Despite analytical considerations of firm size and ownership, we did not find evidence to support our prediction. In fact, neither the interacted variable of location in a monotown with asset size nor that with state ownership nor the five pairs of dummy variables for firms with different employment scales show a significant estimate in the extended models. Judging from these supplemental estimation results as well as the findings reported in the previous subsections, we conjecture that, in general, the government both in central and regional levels does not provide any effective policy treatments specific to single-industry municipalities for keeping their companies alive.^14^

## Conclusions

In this paper, using a dataset of 93,260 firms, we traced the survival status of Russian business companies in the period of 2007–2019 and empirically examined the determinants of distressed acquisitions. We found that, of 93,260 firms, 50,743, or 54.4%, were financially distressed, and 10,110, or 19.9%, of failed firms were rescued by acquisition during the observation period. The empirical results indicate that, in Russian regions, the weakness of the legal system is positively associated with the probability of distressed acquisitions, while the socioeconomic risks are negatively related to it. These tendencies are common in most industries and regions. In this sense, our results strongly demonstrated the surprisingly high predictive performance of the initial level of the region-level legal weakness and other investment risks as a factor explaining differences in the frequency of acquisition of financially distressed firms across Russia in the long run; therefore, we reinforced the high validity of the empirical approach of Iwasaki et al. (2021).^15^ Furthermore, it is also revealed that, in the Central and Northwestern Federal Districts, monotown enterprises are more likely to be bailed out by acquisition after management failure than are other firms within the area. However, it is not always true for the whole federation and other regions.

There is a belief that Russian investors and companies intensively acquire distressed firms against the background of an ineffective legal system for bankruptcy and the liquidation of company assets. However, our data exposed that the frequency of distressed acquisition was remarkably lower during the observation period, indicating that bailout by acquisition is no longer a popular means of rescuing failed firms in Russia today. The empirical evidence obtained from this study infers that improvement in the regional arbitrary courts or worsening socioeconomic risks have created the situation observed in the data.

In this regard, we cannot exclude the possibility that the above contradictory developments have proceeded in parallel in recent years. Improvement in the business environment has been declared as a top priority of the Putin administration, and there have been significant attempts at regulatory reform and judicial reform. As pointed out in Iwasaki (2018), there are indications that formal business regulation and practices have progressed significantly in this country. At the same time, the following factors, such as the retreat of democracy under the authoritarian Putin regime, economic stagnation against the backdrop of the global financial crisis, sanctions imposed by Western countries, and the slump in world oil prices, as well as the spread of organized crime and corruption, obviously have greatly increased the investment risk in Russia. These factors have resulted in a sharp increase in firm exits and a slump in firm entries in recent years, as shown in Fig. 1. It is likely that such developments significantly impact investors' decisions regarding the treatment of firms after failure.

Furthermore, contrary to long academic debates and established convictions among a group of experts about the political and economic importance of monotown enterprises, our empirical evidence intimates the policy neutrality of the Russian government toward single-industry municipalities. In other words, from 2007 to 2019, companies in monotowns—regardless of their size and ownership structure—did not enjoy a higher chance of survival and rescue by acquisition as compared with their counterparts located in other cities and towns, *ceteris paribus*. This result implies that Russia might have overcome the negative legacy of socialism to some extent, thanks to progress in the economic transition and some accompanying transformation of the industrial structure during that period.

Thus, our results shed new light for understanding institutional and other determinants of distressed acquisition based on evidence from Russian regions. Although the severity of Russia’s economic recession due to the war in Ukraine is yet to be grasped, we expect increased instances of company failures. This study can, therefore, serve as a reference point for measuring the extent to which legal weakness and socioeconomic risks impact distressed acquisition in Russian regions in the post-war era.

## Appendix 1: Background on the Expert RA Region Rating of Investment Risks

As described in “Data and Empirical Methodology” section, we employ the Expert RA region rating of investment risks in order to empirically test Hypotheses H1 and H2. Expert RA is the leading independent rating agency in Russia and has been rating the constituent entities of the Russian Federation since 1996 (https://www.raexpert.ru/). Expert RA’s regional rankings are the only ratings in Russia that allow comparisons of all federal entities, and are, therefore, used not only for business purposes but also for academic research (Tables

**Table 9:** Survival status and share of distressed acquisitions in failed firms in Russia, federal districts, and regions, 2007–2019 (*Source*: Bureau van Dijk (BvD) Orbis database (https://webhelp.bvdep.com))

	Number of firms operating at the end of 2006 (*N*)	Number of surviving firms (survivors) by the end of 2019 (*A*)	Number of failed firms by the end of 2019				Failure rate (*F*/*N*)	Share of distressed acquisitions in failed firms (*D*/*F*)
			Total failed firms (*F*=*B*+*C*+*D*)	Bankruptcy, liquidation, dissolution (*B*)	Dormant (*C*)	Distressed acquisition (*D*)		
Russian Federation	93,260	42,517	50,743	38,774	1859	10,110	0.544	0.199
Central Federal District	46,485	20,670	25,815	18,968	1082	5765	0.555	0.223
Belgorod Region	796	412	384	297	15	72	0.482	0.188
Bryansk Region	568	292	276	199	10	67	0.486	0.243
Vladimir Region	694	363	331	274	8	49	0.477	0.148
Voronezh Region	1181	563	618	504	23	91	0.523	0.147
Ivanovo Region	539	224	315	246	10	59	0.584	0.187
Kaluga Region	544	320	224	179	13	32	0.412	0.143
Kostroma Region	285	120	165	131	11	23	0.579	0.139
Kursk Region	455	230	225	187	9	29	0.495	0.129
Lipetsk Region	349	178	171	139	2	30	0.490	0.175
Moscow Region	4483	2342	2141	1608	109	424	0.478	0.198
Orel Region	347	188	159	129	10	20	0.458	0.126
Ryazan Region	477	272	205	164	6	35	0.430	0.171
Smolensk Region	529	270	259	207	7	45	0.490	0.174
Tambov Region	238	95	143	126	1	16	0.601	0.112
Tver Region	608	302	306	262	4	40	0.503	0.131
Tula Region	725	380	345	283	21	41	0.476	0.119
Yaroslavl Region	911	411	500	362	31	107	0.549	0.214
Moscow Federal City	32,756	13,708	19,048	13,671	792	4585	0.582	0.241
Northwestern Federal District	9493	4603	4890	4048	149	693	0.515	0.142
Republic of Karelia	451	220	231	195	3	33	0.512	0.143
Republic of Komi	407	196	211	177	7	27	0.518	0.128
Arkhangelsk Region	481	259	222	180	5	37	0.462	0.167
Nenets Autonomous Area	27	15	12	8	0	4	0.444	0.333
Vologda Region	703	321	382	293	7	82	0.543	0.215
Kaliningrad Region	463	252	211	164	6	41	0.456	0.194
Leningrad Region	640	356	284	226	10	48	0.444	0.169
Murmansk Region	493	227	266	215	3	48	0.540	0.180
Novgorod Region	323	191	132	102	8	22	0.409	0.167
Pskov Region	311	174	137	112	4	21	0.441	0.153
St. Petersburg Federal City	5194	2392	2802	2376	96	330	0.539	0.118
Southern Federal District	5614	2764	2850	2168	114	568	0.508	0.199
Republic of Adygeya	74	42	32	28	1	3	0.432	0.094
Republic of Kalmykia	34	11	23	15	1	7	0.676	0.304
Krasnodar Territory	2159	1171	988	765	46	177	0.458	0.179
Astrakhan Region	266	116	150	128	1	21	0.564	0.140
Volgograd Region	922	347	575	429	21	125	0.624	0.217
Rostov Region	2159	1077	1082	803	44	235	0.501	0.217
North Caucasus Federal District	1303	716	587	486	15	86	0.450	0.147
Republic of Daghestan	135	89	46	37	1	8	0.341	0.174
Republic of Ingushetia	n/a	n/a	n/a	n/a	n/a	n/a	n/a	n/a
Kabardino–Balkarian Republic	89	43	46	36	2	8	0.517	0.174
Karachayevo–Circassian Republic	95	54	41	36	1	4	0.432	0.098
Republic of North Ossetia–Alania	121	63	58	49	2	7	0.479	0.121
Chechen Republic	n/a	n/a	n/a	n/a	n/a	n/a	n/a	n/a
Stavropol Territory	863	467	396	328	9	59	0.459	0.149
Volga Federal District	12,678	5892	6786	5224	188	1374	0.535	0.202
Republic of Bashkortostan	1116	544	572	469	17	86	0.513	0.150
Republic of Mari El	310	155	155	123	7	25	0.500	0.161
Republic of Mordovia	298	142	156	124	10	22	0.523	0.141
Republic of Tatarstan	1327	628	699	520	8	171	0.527	0.245
Republic of Udmurtia	774	298	476	408	12	56	0.615	0.118
Chuvash Republic	524	314	210	166	10	34	0.401	0.162
Perm Territory	1249	617	632	500	28	104	0.506	0.165
Kirov Region	703	305	398	282	10	106	0.566	0.266
Nizhny Novgorod Region	1882	924	958	745	27	186	0.509	0.194
Orenburg Region	341	146	195	160	6	29	0.572	0.149
Penza Region	589	279	310	232	11	67	0.526	0.216
Samara Region	1991	825	1166	816	24	326	0.586	0.280
Saratov Region	966	470	496	427	9	60	0.513	0.121
Ulyanovsk Region	608	245	363	252	9	102	0.597	0.281
Ural Federal District	6394	2948	3446	2768	97	581	0.539	0.169
Kurgan Region	254	121	133	114	4	15	0.524	0.113
Sverdlovsk Region	2409	1048	1361	1069	25	267	0.565	0.196
Tyumen Region	832	359	473	375	25	73	0.569	0.154
Khanty–Mansi Autonomous Area–Yugra	926	507	419	328	11	80	0.452	0.191
Yamal–Nenets Autonomous Area	242	112	130	109	0	21	0.537	0.162
Chelyabinsk Region	1731	801	930	773	32	125	0.537	0.134
Siberian Federal District	8035	3272	4763	3832	145	786	0.593	0.165
Republic of Altai	147	29	118	82	6	30	0.803	0.254
Republic of Tuva	23	12	11	11	0	0	0.478	0.000
Republic of Khakasia	155	74	81	70	2	9	0.523	0.111
Altai Territory	949	392	557	465	25	67	0.587	0.120
Krasnoyarsk Territory	1152	546	606	474	21	111	0.526	0.183
Irkutsk Region	1102	473	629	494	12	123	0.571	0.196
Kemerovo Region	1220	437	783	593	16	174	0.642	0.222
Novosibirsk Region	1,843	693	1,150	943	33	174	0.624	0.151
Omsk Region	762	289	473	409	14	50	0.621	0.106
Tomsk Region	682	327	355	291	16	48	0.521	0.135
Far East Federal District	3258	1652	1606	1280	69	257	0.493	0.160
Republic of Buryatia	175	89	86	75	4	7	0.491	0.081
Republic of Sakha (Yakutia)	322	177	145	129	6	10	0.450	0.069
Zabaikalsk Territory	143	71	72	64	1	7	0.503	0.097
Kamchatka Territory	210	91	119	99	2	18	0.567	0.151
Primorsky Territory	980	496	484	382	25	77	0.494	0.159
Khabarovsk Territory	765	373	392	283	21	88	0.512	0.224
Amur Region	202	100	102	91	4	7	0.505	0.069
Magadan Region	137	66	71	57	2	12	0.518	0.169
Sakhalin Region	288	166	122	87	4	31	0.424	0.254
Jewish Autonomous Region	26	17	9	9	0	0	0.346	0.000
Chukotka Autonomous Area	10	6	4	4	0	0	0.400	0.000

^9^,

**Table 10:** Estimation results of the principal component analysis of region-level risk variables

Eigenvalue of the correlation matrix				Eigenvectors of the first component	
Component no.	Eigenvalue	Difference	Cumulative percentage of total variance	Variables	Eigenvector
1	2.4125	1.204	0.483	Economic risk	0.5214
2	1.2081	0.607	0.242	Financial risk	0.5426
3	0.6009	0.149	0.120	Criminal risk	0.4476
4	0.4521	0.126	0.090	Political risk	0.0063
5	0.3264	–	0.065	Administrative risk	0.4830

For sources, definitions, and descriptive statistics of the variables, see Table 3

^10^,

**Table 11:** Correlation matrix of variables used in empirical analysis

Variable no.	Variable name	[1]	[2]	[3]	[4]	[5]	[6]	[7]	[8]	[9]	[10]	[11]	[12]	[13]	[14]
[1]	Legal weakness	1.000													
[2]	Economic risk	− 0.483	1.000												
[3]	Financial risk	− 0.380	0.593	1.000											
[4]	Criminal risk	− 0.450	0.491	0.389	1.000										
[5]	Political risk	0.236	− 0.127	0.019	− 0.139	1.000									
[6]	Administrative risk	− 0.232	0.404	0.585	0.341	0.267	1.000								
[7]	Comprehensive socioeconomic risk	− 0.496	0.810	0.843	0.695	0.011	0.750	1.000							
[8]	Location in a monotown	− 0.035	0.123	0.172	0.116	− 0.068	0.039	0.146	1.000						
[9]	Open joint-stock company	− 0.028	0.042	0.055	0.009	− 0.017	0.035	0.047	0.027	1.000					
[10]	Closed joint-stock company	0.017	− 0.046	− 0.052	− 0.020	0.015	− 0.038	− 0.051	− 0.012	− 0.139	1.000				
[11]	Limited liability company	− 0.035	0.021	0.021	0.049	− 0.020	0.011	0.032	− 0.019	− 0.495	− 0.592	1.000			
[12]	Large shareholding	0.013	− 0.039	− 0.053	− 0.036	0.015	− 0.039	− 0.054	− 0.038	− 0.032	0.015	0.034	1.000		
[13]	Foreign ownership	0.002	− 0.007	− 0.013	− 0.007	0.004	− 0.006	− 0.011	0.002	0.010	0.015	− 0.006	0.031	1.000	
[14]	Federal state ownership	− 0.005	0.020	0.017	0.008	− 0.007	0.012	0.019	− 0.005	0.177	− 0.020	− 0.147	0.045	− 0.013	1.000
[15]	Regional state ownership	− 0.032	0.053	0.043	0.024	− 0.038	0.017	0.045	0.041	0.131	− 0.063	− 0.247	0.059	− 0.017	− 0.025
[16]	Managerial discretion	− 0.013	− 0.002	0.006	0.014	− 0.003	− 0.021	− 0.001	− 0.006	− 0.003	0.132	− 0.083	− 0.007	− 0.076	− 0.108
[17]	Number of board directors	− 0.040	0.044	0.061	0.022	− 0.021	0.042	0.055	0.025	0.710	− 0.086	− 0.363	0.056	0.017	0.157
[18]	International audit firm	0.001	− 0.003	0.001	0.003	0.002	0.001	0.000	0.006	0.047	− 0.002	− 0.037	0.001	0.068	0.027
[19]	Large Russian audit firm	− 0.017	0.008	0.016	0.009	− 0.004	0.012	0.014	0.001	0.079	− 0.009	− 0.044	0.010	0.011	0.137
[20]	Local Russian audit firm	− 0.021	0.019	0.029	0.014	− 0.008	0.015	0.025	0.012	0.196	− 0.010	− 0.116	0.012	0.026	0.043
[21]	ROA	− 0.041	0.021	− 0.001	0.023	− 0.024	− 0.012	0.010	0.008	− 0.076	0.003	0.099	0.144	0.005	− 0.010
[22]	Gross margin	0.005	− 0.007	− 0.021	0.006	− 0.001	− 0.009	− 0.011	− 0.023	− 0.013	0.043	0.012	0.089	0.038	0.006
[23]	Listing on the stock market	− 0.018	0.018	0.019	0.010	− 0.012	0.007	0.017	0.008	0.216	− 0.033	− 0.118	0.025	0.028	0.112
[24]	Gearing	− 0.024	0.035	0.053	0.029	− 0.010	0.040	0.051	0.002	− 0.004	0.003	0.047	− 0.072	0.011	− 0.040
[25]	Firm size	0.033	− 0.022	− 0.028	− 0.012	− 0.015	− 0.021	− 0.027	− 0.004	0.249	0.122	− 0.296	− 0.076	0.089	0.124
[26]	Firm age	− 0.044	0.050	0.040	0.015	− 0.038	0.008	0.037	0.023	0.310	0.156	− 0.433	0.034	0.008	0.098
[27]	Business network	0.003	− 0.006	− 0.001	− 0.003	− 0.001	− 0.002	− 0.004	0.000	0.166	0.046	− 0.163	0.050	0.021	0.074
[28]	Business diversification	0.191	− 0.134	− 0.151	− 0.143	0.037	− 0.108	− 0.172	− 0.052	− 0.033	0.030	0.024	0.012	− 0.010	− 0.010

Variable no.	Variable name	[15]	[16]	[17]	[18]	[19]	[20]	[21]	[22]	[23]	[24]	[25]	[26]	[27]	[28]
[15]	Regional state ownership	1.000													
[16]	Managerial discretion	− 0.153	1.000												
[17]	Number of board directors	0.086	− 0.001	1.000											
[18]	International audit firm	− 0.005	− 0.014	0.041	1.000										
[19]	Large Russian audit firm	− 0.004	− 0.014	0.118	− 0.001	1.000									
[20]	Local Russian audit firm	− 0.007	− 0.016	0.237	− 0.002	− 0.003	1.000								
[21]	ROA	− 0.044	0.027	− 0.030	0.009	0.000	− 0.011	1.000							
[22]	Gross margin	− 0.041	0.004	0.012	0.033	0.007	0.015	0.338	1.000						
[23]	Listing on the stock market	− 0.003	− 0.020	0.295	0.157	0.194	0.288	0.003	0.032	1.000					
[24]	Gearing	− 0.056	− 0.021	− 0.003	− 0.004	0.001	0.006	− 0.176	− 0.032	− 0.005	1.000				
[25]	Firm size	0.056	− 0.072	0.245	0.096	0.061	0.139	− 0.193	0.030	0.177	0.148	1.000			
[26]	Firm age	0.111	0.089	0.268	0.034	0.045	0.132	0.001	0.063	0.121	− 0.035	0.232	1.000		
[27]	Business network	0.018	0.022	0.195	0.220	0.052	0.134	− 0.008	0.048	0.246	0.012	0.237	0.161	1.000	
[28]	Business diversification	− 0.044	− 0.013	− 0.014	− 0.021	− 0.009	− 0.023	− 0.026	− 0.001	− 0.027	0.004	0.081	− 0.084	0.021	1.000

For sources, definitions, and descriptive statistics of the variables, see Table 3

^11^,

**Table 12:** Univariate comparison of surviving firms and failed firms

Variable name	Survival status at the end of 2019				Univariate comparison	
	Surviving firms		Failed firms		Test for equality of means (*t*) or test for equality of proportions (*z*)	Wilcoxon rank-sum test (*z*)
	Mean	Median	Mean	Median		
*Region-level variables*						
Location in a monotown	0.060	0	0.057	0	1.548	1.548
*Firm-level control variables*						
Open joint-stock company	0.137	0	0.076	0	30.081***	30.080***
Closed joint-stock company	0.166	0	0.123	0	18.436***	18.435***
Limited liability company	0.606	1	0.743	1	− 44.079***	− 44.078***
Large shareholding	0.991	1	0.832	1	81.956***	81.956***
Foreign ownership	0.012	0	0.007	0	7.227***	7.227***
Federal state ownership	0.026	0	0.013	0	14.199***	14.199***
Regional state ownership	0.040	0	0.024	0	14.175***	14.175***
Managerial discretion	3.543	5	3.336	0	7.824***	7.255***
Board size	1.766	1	1.251	1	42.285***	44.070***
International audit firm	0.0012	0	0.0002	0	6.066***	6.066***
Large Russian audit firm	0.0018	0	0.0003	0	7.217***	7.217***
Local Russian audit firm	0.011	0	0.003	0	13.264***	13.264***
ROA	13.824	9	7.587	4	46.689***	60.867v
Gross margin	15.818	12.680	11.451	7.380	35.513***	57.629***
Listing on the stock market	0.011	0.000	0.002	0.000	16.272***	16.272***
Gearing	64.863	3.810	85.929	0.610	− 17.464***	8.387***
Firm size	10.230	10.141	9.929	9.926	27.033***	22.867***
Firm age	2.103	2	1.671	2	86.561***	85.463***
Business network	0.998	0	0.366	0	30.204***	64.250***
Business diversification	6.729	7	6.873	7	− 5.794***	− 6.394***

*** denotes statistical significance at the 1% level. Table 3 provides definitions and descriptive statistics of variables

12,

**Table 13:** Determinants of firm distress: estimation results of the first stage of a Heckman two-stage probit analysis with sample selection

Target industry/sample restriction/region group	All industries	Agriculture, forestry, and fishing (Section A)	Mining, energy, and manufacturing (Sections B–E)	Construction (Section F)	Nonfinancial services (Sections G–J, L–S)	Financial services (Section K)	Central and Northwestern Federal Districts	Southern and North Caucasus Federal Districts
Model	Table 5 Model [1]	Table 6 Model [1]	Table 6 Model [3]	Table 6 Model [5]	Table 6 Model [7]	Table 6 Model [9]	Table 7 Model [1]	Table 7 Model [3]
Location in a monotown	− 0.00883 (0.0233)	0.08145 (0.1713)	0.00188 (0.0400)	0.00372 (0.0624)	− 0.02205 (0.0330)	0.35112 (0.4974)	− 0.09395* (0.0542)	− 0.02108 (0.1385)
Location in a monotown × Firm size								
Firms with less than 500 employees in monotowns								
Firms with 500–999 employees in monotowns								
Firms with 1000–4999 employees in monotowns								
Firms with 5000–9999 employees in monotowns								
Firms with 10000 or more employees in monotowns								
Location in a monotown × Federal state ownership								
Location in a monotown × Regional state ownership								
Open joint-stock company	0.11989*** (0.0347)	0.27948*** (0.1050)	− 0.04862 (0.0629)	0.01154 (0.1133)	0.05176 (0.0582)	0.25888 (1.8100)	0.13458*** (0.0419)	0.06757 (0.1509)
Closed joint-stock company	0.03576 (0.0289)	0.22167*** (0.0665)	− 0.21390*** (0.0597)	− 0.13823 (0.1032)	0.15382*** (0.0456)	1.10701 (1.7262)	0.06766** (0.0345)	− 0.16705 (0.1325)
Limited liability company	0.07240*** (0.0272)	0.12837* (0.0681)	− 0.18184*** (0.0576)	− 0.07266 (0.1003)	0.18948*** (0.0422)	0.80429 (1.7173)	0.13210*** (0.0326)	− 0.16318 (0.1216)
Large shareholding	− 2.08197*** (0.0817)	− 2.38015*** (0.2458)	− 1.98538*** (0.1345)	− 1.93356*** (0.2148)	− 2.12917*** (0.1411)	− 1.37802** (0.6524)	− 1.96381*** (0.1001)	− 6.60536*** (0.2554)
Foreign ownership	− 0.02659 (0.0538)	0.11240 (0.2654)	− 0.10150 (0.0845)	− 0.14970 (0.2835)	0.02505 (0.0768)	− 0.00564 (0.6062)	− 0.10636* (0.0635)	− 0.11031 (0.2470)
Federal state ownership	0.23640*** (0.0374)	0.44386*** (0.1212)	0.21036*** (0.0576)	0.02023 (0.1220)	0.19651*** (0.0618)	0.47758 (0.8075)	0.29945*** (0.0502)	0.06563 (0.1326)
Regional state ownership	0.17948*** (0.0330)	0.23564** (0.1123)	− 0.05666 (0.0578)	0.17024* (0.1022)	0.32323*** (0.0510)	− 0.80315 (1.3700)	0.27301*** (0.0448)	− 0.01739 (0.1243)
Managerial discretion	0.00450*** (0.0012)	− 0.01515** (0.0062)	− 0.00872*** (0.0033)	0.01051*** (0.0031)	0.01023*** (0.0015)	− 0.00658 (0.0332)	0.00611*** (0.0016)	− 0.00559 (0.0065)
Board size	− 0.04847*** (0.0090)	− 0.02203 (0.0458)	− 0.05871*** (0.0126)	− 0.07122*** (0.0250)	− 0.03859** (0.0164)	− 0.71201** (0.3159)	− 0.03141*** (0.0103)	− 0.06674* (0.0404)
Board size^2^	0.00197*** (0.0006)	− 0.00248 (0.0040)	0.00212*** (0.0008)	0.00441** (0.0018)	0.00264** (0.0013)	0.05492** (0.0252)	0.00107 (0.0007)	0.00181 (0.0029)
International audit firm	0.16699 (0.2232)		0.15114 (0.2542)		− 0.55419 (0.6637)		0.38551 (0.2758)	
Large Russian audit firm	0.01620 (0.1507)	− 0.51997 (0.7199)	− 0.00908 (0.2066)	− 0.05306 (0.5920)	0.25134 (0.2843)	0.99899 (0.7900)	− 0.40024 (0.2573)	0.34730 (0.5294)
Local Russian audit firm	0.18651*** (0.0592)	− 0.22143 (0.3528)	0.17872** (0.0740)	0.02442 (0.2770)	0.08098 (0.1371)	− 0.00307 (0.0077)	0.24577*** (0.0826)	− 0.59112 (0.5026)
ROA	− 0.00709*** (0.0003)	− 0.00663*** (0.0023)	− 0.00627*** (0.0007)	− 0.00539*** (0.0008)	− 0.00774*** (0.0004)	− 0.01268*** (0.0048)	− 0.00717*** (0.0004)	− 0.00463*** (0.0011)
Gross margin	− 0.00273*** (0.0004)	− 0.00719*** (0.0018)	− 0.00413*** (0.0008)	− 0.00532*** (0.0010)	− 0.00076* (0.0005)	0.11473 (0.2940)	− 0.00334*** (0.0004)	− 0.00163 (0.0014)
Listing on the stock market	0.15364** (0.0736)	0.59362 (0.5158)	0.06012 (0.0903)	0.62087*** (0.1983)	− 0.46856* (0.2444)	− 0.00020 (0.0005)	0.20410** (0.1041)	− 0.03757 (0.2372)
Gearing	0.00026*** (0.0000)	0.00082*** (0.0002)	0.00041*** (0.0001)	0.00028*** (0.0001)	0.00018*** (0.0000)	− 0.00394 (0.0806)	0.00027*** (0.0000)	0.00024** (0.0001)
Firm size	− 0.05833*** (0.0041)	− 0.12579*** (0.0206)	− 0.07260*** (0.0085)	− 0.04295*** (0.0106)	− 0.05367*** (0.0055)	− 0.01580 (0.1777)	− 0.05760*** (0.0051)	− 0.05102*** (0.0171)
Firm age	− 0.40813*** (0.0082)	− 0.21277*** (0.0421)	− 0.30741*** (0.0164)	− 0.41191*** (0.0228)	− 0.46901*** (0.0110)	0.01103 (0.0322)	− 0.42650*** (0.0107)	− 0.32150*** (0.0318)
Business network	− 0.01647*** (0.0026)	− 0.00460 (0.0094)	− 0.00917** (0.0046)	− 0.03324*** (0.0077)	− 0.01854*** (0.0039)	0.02272 (0.0312)	− 0.01878*** (0.0032)	− 0.02420* (0.0128)
Business diversification	0.00246* (0.0015)	− 0.00337 (0.0051)	0.00797*** (0.0029)	0.00500 (0.0042)	0.00072 (0.0020)	1.41285 (2.0220)	0.00582*** (0.0019)	− 0.00126 (0.0055)
NACE division-level fixed effects	Yes	Yes	Yes	Yes	Yes	Yes	Yes	Yes

Target industry/sample restriction/region group	Volga and Ural Federal Districts	Siberian and Far East Federal Districts	Without Moscow Federal City, Moscow Region, St. Petersburg Federal City, and Leningrad Region	All industries			Without companies that failed in 2007–2009	Without companies that failed in 2007–2012	Without companies that failed in 2007–2015
Model	Table 7 Model [5]	Table 7 Model [7]	Table 7 Model [9]	Table 8 Model [1]	Table 8 Model [3]	Table 8 Model [5]	Appendix Table 14 Model [1]	Appendix Table 14 Model [3]	Appendix Table 14 Model [5]
Location in a monotown	− 0.03718 (0.0330)	0.20788*** (0.0511)	0.04265* (0.0239)	− 0.03829 (0.1508)		− 0.01736 (0.0245)	− 0.01490 (0.0237)	0.01239 (0.0245)	− 0.03323 (0.0307)
Location in a monotown × Firm size				0.00293 (0.0149)					
Firms with less than 500 employees in monotowns					− 0.00934 (0.0238)				
Firms with 500–999 employees in monotowns					− 0.08315 (0.1424)				
Firms with 1000–4999 employees in monotowns					0.06665 (0.2033)				
Firms with 5000–9999 employees in monotowns					0.19079 (0.3795)				
Firms with 10000 or more employees in monotowns					0.30327 (0.4306)				
Location in a monotown × Federal state ownership						0.14336 (0.1579)			
Location in a monotown × Regional state ownership						0.07387 (0.0880)			
Open joint-stock company	0.01414 (0.0842)	− 0.02793 (0.1223)	0.03573 (0.0508)	0.12004*** (0.0347)	0.11981*** (0.0347)	0.11955*** (0.0347)	0.13711*** (0.0368)	0.10537*** (0.0376)	0.10338** (0.0485)
Closed joint-stock company	− 0.08510 (0.0754)	− 0.02345 (0.1052)	− 0.10848** (0.0450)	0.03576 (0.0289)	0.03589 (0.0289)	0.03572 (0.0289)	0.06086** (0.0293)	0.09901*** (0.0307)	0.09681** (0.0386)
Limited liability company	− 0.08664 (0.0713)	− 0.05720 (0.0982)	− 0.08361** (0.0420)	0.07239*** (0.0272)	0.07234*** (0.0272)	0.07230*** (0.0272)	0.06986** (0.0277)	0.08928*** (0.0290)	0.11193*** (0.0364)
Large shareholding	− 2.36003*** (0.2313)	− 1.95319*** (0.2067)	− 2.21074*** (0.1155)	− 2.08191*** (0.0817)	− 2.08271*** (0.0817)	− 2.08229*** (0.0817)	− 2.09494*** (0.0841)	− 1.58868*** (0.1035)	− 0.43140** (0.1927)
Foreign ownership	0.22859* (0.1355)	0.16989 (0.2166)	0.08748 (0.0781)	− 0.02657 (0.0538)	− 0.02759 (0.0539)	− 0.02666 (0.0538)	− 0.06846 (0.0550)	− 0.17001*** (0.0599)	− 0.08844 (0.0729)s
Federal state ownership	0.21899*** (0.0822)	0.07990 (0.1048)	0.19603*** (0.0487)	0.23656*** (0.0374)	0.23576*** (0.0374)	0.22881*** (0.0383)	0.21419*** (0.0379)	0.19938*** (0.0393)	0.17875*** (0.0492)
Regional state ownership	0.08089 (0.0694)	− 0.09043 (0.1033)	0.04351 (0.0436)	0.17951*** (0.0330)	0.17930*** (0.0330)	0.17178*** (0.0342)	0.12288*** (0.0338)	0.07056** (0.0352)	0.01898 (0.0444)
Managerial discretion	0.00510** (0.0024)	− 0.00960** (0.0047)	0.00229 (0.0015)	0.00450*** (0.0012)	0.00448*** (0.0012)	0.00450*** (0.0012)	− 0.00767*** (0.0017)	− 0.01178*** (0.0015)	− 0.00947*** (0.0018)
Board size	− 0.07661*** (0.0178)	− 0.05322** (0.0266)	− 0.05781*** (0.0114)	− 0.04850*** (0.0090)	− 0.04835*** (0.0090)	− 0.04829*** (0.0090)	− 0.04467*** (0.0106)	− 0.01940* (0.0100)	− 0.00977 (0.0142)
Board size^2^	0.00396*** (0.0011)	0.00347** (0.0016)	0.00261*** (0.0007)	0.00197*** (0.0006)	0.00195*** (0.0006)	0.00196*** (0.0006)	0.00168** (0.0007)	0.00072 (0.0007)	− 0.00004 (0.0010)
International audit firm	− 0.70038 (0.4562)	− 0.47358 (0.3227)	− 0.11070 (0.3225)	0.16681 (0.2232)	0.15818 (0.2239)	0.16718 (0.2234)	− 0.00922 (0.2773)	− 0.09070 (0.2837)	− 3.94919 (207.3188)s
Large Russian audit firm	0.36394 (0.2410)	− 0.29750 (0.4975)	− 0.03428 (0.1462)	0.01033 (0.1515)	− 0.00438 (0.1554)	0.01654 (0.1510)	− 0.20844 (0.1936)	− 0.27831 (0.1923)	− 0.35263 (0.2715)
Local Russian audit firm	0.01752 (0.0971)	0.03091 (0.1924)	0.11948* (0.0654)	0.18613*** (0.0593)	0.18537*** (0.0595)	0.18676*** (0.0593)	0.16031** (0.0699)	0.06425 (0.0661)	0.11157 (0.0858)s
ROA	− 0.00706*** (0.0007)	− 0.00793*** (0.0009)	− 0.00639*** (0.0004)	− 0.00709*** (0.0003)	− 0.00709*** (0.0003)	− 0.00708*** (0.0003)	− 0.00695*** (0.0003)	− 0.00669*** (0.0003)	− 0.00583*** (0.0004)
Gross margin	− 0.00295*** (0.0009)	0.00061 (0.0011)	− 0.00209*** (0.0005)	− 0.00273*** (0.0004)	− 0.00273*** (0.0004)	− 0.00273*** (0.0004)	− 0.00275*** (0.0004)	− 0.00292*** (0.0004)	− 0.00226*** (0.0005)
Listing on the stock market	0.37294*** (0.1433)	− 0.26262 (0.2284)	0.06126 (0.0884)	0.15429** (0.0736)	0.15087** (0.0739)	0.15356** (0.0736)	0.17599** (0.0751)	0.06228 (0.0802)	− 0.07015 (0.1093)
Gearing	0.00025*** (0.0001)	0.00045*** (0.0001)	0.00032*** (0.0000)	0.00026*** (0.0000)	0.00026*** (0.0000)	0.00026*** (0.0000)	0.00029*** (0.0000)	0.00033*** (0.0000)	0.00024*** (0.0000)
Firm size	− 0.06726*** (0.0096)	− 0.06753*** (0.0130)	− 0.05371*** (0.0059)	− 0.05850*** (0.0042)	− 0.05839*** (0.0041)	− 0.05830*** (0.0041)	− 0.06434*** (0.0042)	− 0.06981*** (0.0044)	− 0.07469*** (0.0055)
Firm age	− 0.37613*** (0.0182)	− 0.38109*** (0.0248)	− 0.35809*** (0.0112)	− 0.40813*** (0.0082)	− 0.40812*** (0.0082)	− 0.40810*** (0.0082)	− 0.38699*** (0.0084)	− 0.35535*** (0.0088)	− 0.29374*** (0.0109)
Business network	− 0.00874 (0.0058)	− 0.01629** (0.0081)	− 0.01307*** (0.0036)	− 0.01646*** (0.0026)	− 0.01653*** (0.0026)	− 0.01646*** (0.0026)	− 0.01448*** (0.0026)	− 0.01049*** (0.0026)	− 0.00295 (0.0030)
Business diversification	− 0.01199*** (0.0033)	0.00001 (0.0044)	− 0.00525*** (0.0020)	0.00246* (0.0015)	0.00246* (0.0015)	0.00245* (0.0015)	0.00232 (0.0015)	0.00184 (0.0016)	0.00309 (0.0019)
NACE division-level fixed effects	Yes	Yes	Yes	Yes	Yes	Yes	Yes	Yes	Yes

This table contains estimation results of the first stage of a Heckman two-stage probit model with a sample selection of the determinants of distressed acquisition. The coefficient of the constant term is omitted from the table. The dependent variable is a dummy variable for failed firms. The estimation results of the second stage are reported in Tables 5, 6, 7, 8 and Appendix Table 14. Table 3 provides detailed definitions and descriptive statistics of the independent variables used in the estimation. Figures in parentheses are robust standard errors

***, **, and * denote statistical significance at the 1%, 5%, and 10% levels, respectively

13).

According to Expert (2006), various quantitative and qualitative indices were utilized to assess the components of investment risks in the economy, finance, crime, politics, and administration by region. Their main information sources are data obtained from Rosstat, the Ministry of Finance, the Ministry of Economic Development and Trade, the Ministry of Regional Development, the Ministry of Information Technology and Communication, the Central Bank of Russia, the Ministry for Taxes and Levies, the Ministry of Natural Resources, the Ministry of Interior, as well as the database of Expert RA rating agency and news feeds of the Russian press.

To assess the investment risk in the legal system by region, the legislative reference system operated by Consultant Plus was used. Consultant Plus was established in 1992 as a consulting agency for experts whose work is connected to the application of legislation, including lawyers, accountants, and the staff of budgetary organizations. It also runs the largest Internet-based legal information database in Russia that contains documents of federal and regional legislation, court decisions, financial advice, legislative comments, and other legal information (http://www.consultant.ru/). When compiling the rating, information on legislation presented on the websites of the regions as well as information sent by administrators of the Federal entities was also considered.

It is unfortunate that Expert RA discontinued publication of investment risk ratings in politics and the legal system in 2006 and 2010, respectively. Ratings of investment risks in the economy, finance, crime, and administration were being published until 2020, in addition to those of social and ecological investment risks.

Although some regions show remarkable rises or falls in their investment risk rankings during the observation period, the interregional relationship is stable over time. In fact, the Kendall rank correlation coefficients between the 2006 and 2018 ratings for economic, financial, crime, and administrative investment risk are all around 0.50 and statistically significant at the 0.1% level. That is one reason for employing the Expert RA region rating to test Hypotheses H1 and H2 in this paper. The high predictive power of investment risk variables in the long run is demonstrated in Appendix Table

**Table 14:** Determinants of distressed acquisition: estimation with periodic sample restrictions

Periodic sample restriction	Without companies that failed in 2007–2009		Without companies that failed in 2007–2012		Without companies that failed in 2007–2015	
Model	[1]	[2]	[3]	[4]	[5]	[6]
*Region-level variables*						
Legal weakness	0.00279***		0.00082***		0.00098**	
	(0.0005)		(0.0003)		(0.0005)	
Comprehensive socioeconomic risk		− 0.03410***		− 0.02724***		− 0.00294
		(0.0074)		(0.0101)		(0.0044)
Location in a monotown	0.01403	0.03725	0.05426	0.08098	0.10172*	0.05500
	(0.0384)	(0.0377)	(0.0337)	(0.0528)	(0.0538)	(0.0338)
*Firm-level control variables*						
Open joint-stock company	− 0.40162***	− 0.38629***	− 0.27025***	− 0.27113***	− 0.34437***	− 0.35263***
	(0.0707)	(0.0745)	(0.0517)	(0.0515)	(0.0894)	(0.0902)
Closed joint-stock company	− 0.21568***	− 0.21994***	− 0.19382***	− 0.19603***	− 0.31563***	− 0.31396***
	(0.0531)	(0.0530)	(0.0446)	(0.0445)	(0.0825)	(0.0818)
Limited liability company	− 0.18075***	− 0.18769***	− 0.20390***	− 0.20724***	− 0.35766***	− 0.36240***
	(0.0489)	(0.0480)	(0.0426)	(0.0426)	(0.0817)	(0.0818)
Large shareholding	1.58926***	1.64364***	1.42736***	1.43118***	2.43265	2.45285
	(0.2475)	(0.2376)	(0.1209)	(0.1202)	(73.8645)	(81.8907)
Foreign ownership	0.36964***	0.35456***	0.34100***	0.33822***	0.33166***	0.34261***
	(0.0909)	(0.0911)	(0.0819)	(0.0815)	(0.1286)	(0.1295)
Federal state ownership	0.22685**	0.19607*	0.04967	0.04325	0.20185*	0.19767
	(0.1041)	(0.1059)	(0.0651)	(0.0642)	(0.1219)	(0.1206)
Regional state ownership	0.39882***	0.36987***	0.19747***	0.19203***	0.30518***	0.29579***
	(0.1008)	(0.1051)	(0.0618)	(0.0610)	(0.1056)	(0.1037)
ROA	0.00453***	0.00477***	0.00703***	0.00700***	0.00791***	0.00790***
	(0.0012)	(0.0011)	(0.0004)	(0.0004)	(0.0008)	(0.0008)
Gross margin	0.00270***	0.00285***	0.00326***	0.00328***	0.00316***	0.00314***
	(0.0006)	(0.0006)	(0.0005)	(0.0005)	(0.0008)	(0.0008)
Listing on the stock market	− 0.14975	− 0.16419	− 0.26958*	− 0.26817*	− 0.21085	− 0.21146
	(0.1522)	(0.1470)	(0.1454)	(0.1444)	(18.9079)	(20.6294)
Gearing	− 0.00033***	− 0.00033***	− 0.00037***	− 0.00038***	− 0.00037***	− 0.00038***
	(0.0001)	(0.0001)	(0.0000)	(0.0000)	(0.0001)	(0.0001)
Firm size	0.10133***	0.10148***	0.10173***	0.10136***	0.16285***	0.16365***
	(0.0067)	(0.0062)	(0.0070)	(0.0070)	(0.0237)	(0.0237)
Firm age	0.07674	0.09617	0.27093***	0.27024***	0.22981***	0.22828***
	(0.0788)	(0.0786)	(0.0213)	(0.0212)	(0.0330)	(0.0331)
Business network	− 0.01339	− 0.01144	− 0.00069	− 0.00056	− 0.01251	− 0.01276
	(0.0087)	(0.0087)	(0.0049)	(0.0049)	(0.0082)	(0.0083)
Business diversification	0.00739***	0.00878***	0.00025	0.00121	− 0.00739**	− 0.00719**
	(0.0028)	(0.0030)	(0.0022)	(0.0022)	(0.0037)	(0.0037)
NACE division-level fixed effects	Yes	Yes	Yes	Yes	Yes	Yes
N	59,303	59,303	54,325	54325	43,193	43,193
Censored observations	25,320	25,320	20,342	20,342	9210	9210
Uncensored observations	33,983	33,983	33,983	33,983	33,983	33,983
Log likelihood	− 48344.300	− 48366.680	− 39937.470	− 39943.110	− 22720.800	− 22717.390
Wald test (*χ*^2^)	1434.14***	1506.50***	2935.54***	2944.46***	927.19***	925.65***
*ρ*	− 0.527	− 0.594	− 0.902	− 0.904	− 0.906	− 0.906
LR test (*χ*^2^)	3.45*	4.30**	30.16***	30.98***	9.90***	10.02***

This table contains estimation results of a Heckman probit model with a sample selection of the determinants of distressed acquisition. The coefficient of the constant term is omitted from the table. The estimation results of the first stage are reported in Appendix Table 13. Table 3 provides detailed definitions and descriptive statistics of the independent variables used in the estimation. Figures in parentheses are robust standard errors. The Wald test examines the null hypothesis that all coefficients are zero. The LR test of the independence of equations examines the null hypothesis that *ρ* = 0

***, **, and * denote statistical significance at the 1%, 5%, and 10% levels, respectively

14, which reports the estimation results using a sample of failed firms, subject to periodic restrictions. In this table, the variable of comprehensive socioeconomic risk is estimated to be insignificant only in Model [6] without a sample of firms that failed from 2007 to 2015, while the variable of legal weakness shows a significant and positive estimate in all the three models. It is noteworthy that firm-level control variables also display significant estimates in line with those in the baseline estimation in Table 5.

## Appendix 2: Estimation Using Alternative Region-Level Variables

In this appendix, we examine whether regional factors other than legal weakness and other socio-economic investment risks affect the likelihood of distressed acquisitions of Russian firms.

Here we turn our attention to the next six factors—(a) macroeconomic performance, (b) firm population density, (c) access to finance, (d) financial soundness of the corporate sector, (e) the size of government, and (f) the size of the judicial sector—assuming that Russian firms tend to be rescued by acquisition after financial distress more frequently in regions with better economic performance, greater opportunity for contacts between corporations, and a corporate sector that is more financially sound, while distressed acquisitions take place less in regions with easier access to finance and stronger administrative and judicial sectors.

The variables that are proxies for the above regional factors are listed in Table 3. We estimated these alternative region-level variables using the same method as in the previous subsections. Appendix Table

**Table 15:** Determinants of distressed acquisition: estimation using alternative region-level variables

Model	[1]	[2]	[3]	[4]	[5]	[6]
*Alternative region-level variables*						
Economic growth	0.01259***					
	(0.0024)					
Firm population density		0.04989***				
		(0.00748)				
Access to finance			− 0.16389***			
			(0.0191)			
Financial soundness of the corporate sector				0.00081		
				(0.0009)		
Government size					− 0.09489***	
					(0.0163)	
Judicial sector size						− 0.07986***
						(0.01444)
Location in a monotown	0.03197	0.04397	0.02883	0.01636	0.02301	0.03138
	(0.0274)	(0.0272)	(0.0273)	(0.0272)	(0.0270)	(0.0271)
*Firm-level control variables*						
Open joint-stock company	− 0.23885***	− 0.23012***	− 0.23381***	− 0.23940***	− 0.23434***	− 0.23381***
	(0.0415)	(0.0404)	(0.0413)	(0.0413)	(0.0408)	(0.0408)
Closed joint-stock company	− 0.16442***	− 0.16298***	− 0.15658***	− 0.16401***	− 0.16425***	− 0.16411***
	(0.0367)	(0.0361)	(0.0367)	(0.0364)	(0.0363)	(0.0363)
Limited liability company	− 0.14039***	− 0.13885***	− 0.13493***	− 0.14438***	− 0.14226***	− 0.14184***
	(0.0340)	(0.0335)	(0.0341)	(0.0338)	(0.0337)	(0.0337)
Large shareholding	1.91731***	1.92001***	1.91109***	1.92363***	1.91996***	1.91929***
	(0.0693)	(0.0682)	(0.0695)	(0.0686)	(0.0686)	(0.0686)
Foreign ownership	0.24300***	0.24082***	0.25244***	0.24214***	0.24357***	0.24200***
	(0.0645)	(0.0639)	(0.0648)	(0.0643)	(0.0642)	(0.0641)
Federal state ownership	− 0.00348	− 0.00693	0.00773	− 0.01310	− 0.00730	− 0.00793
	(0.0481)	(0.0473)	(0.0484)	(0.0475)	(0.0475)	(0.0475)
Regional state ownership	0.15930***	0.15613***	0.16555***	0.15159***	0.15497***	0.15472***
	(0.0455)	(0.0444)	(0.0457)	(0.0449)	(0.0447)	(0.0447)
ROA	0.00571***	0.00582***	0.00579***	0.00572***	0.00577***	0.00576***
	(0.0004)	(0.0004)	(0.0004)	(0.0004)	(0.0004)	(0.0004)
Gross margin	0.00282***	0.00282***	0.00283***	0.00283***	0.00284***	0.00284***
	(0.0004)	(0.0004)	(0.0004)	(0.0004)	(0.0004)	(0.0004)
Listing on the stock market	− 0.15423	− 0.15238	− 0.17105*	− 0.15288	− 0.15755	− 0.15631
	(0.0992)	(0.0976)	(0.1010)	(0.0983)	(0.0983)	(0.0982)
Gearing	− 0.00037***	− 0.00036***	− 0.00037***	− 0.00038***	− 0.00037***	− 0.00037***
	(0.00004)	(0.00004)	(0.00004)	(0.00004)	(0.00004)	(0.00004)
Firm size	0.09142***	0.09110***	0.09265***	0.09112***	0.09127***	0.09099***
	(0.0048)	(0.0048)	(0.0049)	(0.0048)	(0.0048)	(0.0048)
Firm age	0.20649***	0.21348***	0.20720***	0.20705***	0.20959***	0.20976***
	(0.0172)	(0.0159)	(0.0171)	(0.0169)	(0.0165)	(0.0165)
Business network	− 0.00066	− 0.00017	− 0.00113	− 0.00056	− 0.00014	− 0.00005
	(0.0043)	(0.0041)	(0.0043)	(0.0042)	(0.0042)	(0.0042)
Business diversification	0.00428**	0.00351**	0.00300*	0.00546***	0.00418**	0.00423**
	(0.0018)	(0.0017)	(0.0018)	(0.0018)	(0.0018)	(0.0018)
NACE division-level fixed effects	Yes	Yes	Yes	Yes	Yes	Yes
N	61,016	61,016	61,016	61,016	61,016	61,016
Censored observations	27,033	27,033	27,033	27,033	27,033	27,033
Uncensored observations	33,983	33,983	33,983	33,983	33,983	33,983
Log likelihood	− 51634.110	− 51623.140	− 51590.050	− 51649.430	− 51629.580	− 51632.180
Wald test (*χ*^2^)	3090.830***	3233.880***	3267.200***	3058.930***	3175.310***	3162.450***
*ρ*	− 0.929	− 0.936	− 0.926	− 0.933	− 0.934	− 0.934
LR test (*χ*^2^)	33.09***	37.58***	32.82***	35.61***	35.71***	35.69***

This table contains estimation results of a Heckman probit model with a sample selection of the determinants of distressed acquisition. The coefficient of the constant term is omitted from the table. The estimation results of the first stage are omitted from the report. Table 3 provides detailed definitions and descriptive statistics of the independent variables used in the estimation. Figures in parentheses are robust standard errors. The Wald test examines the null hypothesis that all coefficients are zero. The LR test of the independence of equations examines the null hypothesis that *ρ* = 0

***, **, and * denote statistical significance at the 1%, 5%, and 10% levels, respectively

15 shows the results. Although the statistical significance of the variables in question is about 1.5 to 2.5 lower in terms of the *t*-value as compared with our main region-level variables, they are estimated to be significant with a predicted sign except for the variable of financial soundness of the corporate sector. In this table, location in a monotown repeatedly shows an insignificant coefficient, and firm-level control variables display estimates similar to those in Table 5. The results in Appendix Table 15 suggest that distressed acquisitions in Russia are likely influenced by a wide variety of regional factors, thus, requiring further research.

## Footnotes

1 In June 2020, the IMF projected Russia’s real GDP growth rate in 2020 to be − 6.6% (IMF 2020), while the Russian Ministry of Economic Development forecasted a growth rate of − 5% for the same year (https://economy.gov.ru/).

2 Basically, the survival analysis is designed to regress the probability of an event occurring on ex ante conditions. By doing so, this method avoids the endogeneity problem arising from simultaneity of the dependent and independent variables (Iwasaki 2014).

3 The government order in 2014 originally listed 313. After several amendments, the current 2020 version lists 334.

4 We referred to the website of Monotown Development Fund at http://xn--80afd4affbbat.xn--p1ai/

5 Orbis is one of the largest company databases, covering more than 400 million companies worldwide. For details of the database, see BvD’s website: https://webhelp.bvdep.com.

6 Following the precedent of Iwasaki et al. (2021), firms whose status had changed to “merged/taken over” without any notification of management failure in the preceding period are not included in the dataset because these cases may contain peaceful or hostile takeovers of good performing firms that were not triggered by the financial distress of the acquired company.

7 Appendix Table 9 shows the survival status and share of distressed acquisitions by federal constituent entity.

8 See “Appendix 1” for background information regarding the Expert RA region rating of investment risks.

9 The estimation results of the principal component analysis of the five variables are reported in Appendix Table 10.

10 Appendix Table 12 reports a univariate comparison between surviving firms and failed firms. From this table, we verify that the firm-level characteristics significantly differ between the two in line with the arguments in Iwasaki and Kim (2020) and Iwasaki et al. (2021). Meanwhile, it is also found that the surviving and failed firms are not different from the viewpoint of location in a monotown.

11 Dormant firms are totally excluded from the empirical analysis in this section because their final resolution outcome is not specified. For the sake of brevity, Appendix Table 13 shows the first-stage estimation result of Model [1] of Table 5 only. The same reporting policy applies to Tables 6, 7 and 8.

12 It is worth pointing out that the estimation results of the firm-level variables are mostly consistent with those for firms in European emerging markets (Iwasaki et al. 2021).

13 We appreciate a reviewer’s suggestion that a robustness check was needed from this perspective.

14 In “Appendix 2”, we examined the impact of regional factors other than legal weakness and other socio-economic investment risks. The research motivation in the appendix is strongly inspired by two reviewers of the journal. Their helpful comments are acknowledged and highly appreciated.

15 Conversely, while this paper and that of Iwasaki et al. (2021) both emphasize the significance of initial conditions, both works lack any analytical consideration of the effects of time-series changes in the institutional environment surrounding firms on the probability of distressed acquisitions. This issue can only be examined by empirical studies employing panel data. We thank the reviewers for their criticism on this point.

## References

[CR1] Aidis R, Adachi Y (2007). Russia: Firm entry and survival barriers. Economic Systems.

[CR2] Aidis R, Estrin S, Mickiewicz T (2008). Institutions, networks and entrepreneurship development in Russia: A comparative perspective. Journal of Business Venturing..

[CR3] Alekhina, M. 2021. Mezhdu svoim delom i ugolovnym vse men’she raznitsy. *RBK,* May 26.

[CR4] Annunziata A, Agovino M, Mariani A (2019). Sustainability of Italian families’ food practices: Mediterranean diet adherence combined with organic and local food consumption. Journal of Cleaner Production.

[CR6] Bocharov T, Titaev K, Kurkchiyan M, Kubal A (2018). When business goes to court: Arbitrazh courts in Russia. A sociology of justice in Russia.

[CR7] Burger E (2004). Corruption in the Russian arbitrazh courts: Will there be significant progress in the near term?. The International Lawyer.

[CR8] Burger, E., and R. Gitau. 2010. The Russian Anti-corruption campaign: Public relations, politics or substantive change? *Georgetown Public Law Research Paper no. 10-13.*

[CR9] Claessens S, Djankov S, Klapper L (2003). Resolution of corporate distress in East Asia. Journal of Empirical Finance.

[CR10] Commander S (2018). One-company towns: Scale and consequences. IZA World of Labor.

[CR11] Crowley S (2016). Monotowns and the political economy of industrial restructuring in Russia. Post-Soviet Affairs.

[CR12] Dahiya S, Klapper L (2007). Who survives? A cross-country comparison. Journal of Financial Stability..

[CR13] Dumes, V. 2019. Russian business ombudsman Titov complains to Putin about the poor investment climate. *Bne Intellinews*, May 29.

[CR14] Estrin S, Prevezer M (2011). The role of informal institutions in corporate governance: Brazil, Russia, India and China compared. Asia Pacific Journal of Management..

[CR15] Expert (2006). Rating of investment attractiveness of Russian regions: 2005–2006 years. Expert Magazine.

[CR16] Frye T (2017). Property rights and property wrongs: How power, institutions, and norms shape economic conflict in Russia.

[CR17] Gustafsson P (2013). The emergence of the rule of law in Russia. Global Crime.

[CR18] Hendley K, Murrell P, Ryterman R (2000). Law, relationships and private enforcement: Transactional strategies of Russian enterprises. Europe-Asia Studies.

[CR19] Holmes L (2008). Corruption and organized crime in Putin’s Russia. Europe-Asia Studies.

[CR20] IMF (2020). World economic outlook: June 2020.

[CR21] Iwasaki I (2014). Global financial crisis, corporate governance, and firm survival: The Russian experience. Journal of Comparative Economics.

[CR22] Iwasaki I (2018). Corporate governance system and regional heterogeneity: Evidence from East and West Russia. International Journal of the Economics of Business.

[CR23] Iwasaki, I. 2022. How do economic activities spur the COVID-19 pandemic in Russia? A dynamic panel data analysis. *Post-Communist Economies*. (**In press**).

[CR24] Iwasaki I, Kim B-Y (2020). Legal forms, organizational architecture, and firm failure: A large survival analysis of Russian corporations. European Journal of Law & Economics.

[CR25] Iwasaki I, Kočenda E, Shida Y (2021). Distressed acquisitions: Evidence from European emerging markets. Journal of Comparative Economics.

[CR26] Iwasaki I, Kumo K (2020). Determinants of regional fertility in Russia: A dynamic panel data analysis. Post-Communist Economies.

[CR27] Iwasaki I, Maurel M, Meunier B (2016). Firm entry and exit during a crisis period: Evidence from Russian regions. Russian Journal of Economics.

[CR28] Kirdina S, Vernikov A (2013). Evolution of the banking system in the Russian context: An Institutional View. Journal of Economic Issues.

[CR29] Knox, S. 2016. Russia’s ‘monotowns’ face imminent collapse. *Financial Times*, October 7, www.ft.com.

[CR30] Kornia, A. 2020. V Rossii vse strashnee zanimats’ia biznesom. *Vedomosti,* May 25.

[CR31] Kosals L, Maksimova A (2015). Informality, crime and corruption in Russia: A review of recent literature. Theoretical Criminology.

[CR32] Lambert-Mogiliansky A, Sonin K, Zhuravskaya E (2007). Are Russian commercial courts biased? Evidence from a bankruptcy law transplant. Journal of Comparative Economics.

[CR33] Ledeneva A (2006). How Russia really works.

[CR34] Ledeneva A (2013). Can Russia modernise? Sistema, power networks and informal governance.

[CR35] Mirkin Y, Kuznetsova O, Kuznetsov A (2013). The financial depth of emerging markets: The case of Russia. Competition and Change.

[CR36] Moscow Times. 2021. 80% of Russian Business Owners Fear Arbitrary Arrest. May 26.

[CR37] Nesterov, I. 2019. Russia: From monotowns to pluritowns, *Reinventing Cities*, *UNESCO Courier*, April–June 14–16.

[CR38] Oda H (2019). Roshianiokeru Kaishafunsou no Chusaikanousei (Arbitrability of corporate disputes in Russia). Waseda Hougaku.

[CR39] Pomeranz, W., and M. Rojansky. 2016. Russian corruption: The Kremlin fails to tackle its biggest problem. *Kennan Institute Insight & Analysis*, May 16, www.wilsoncenter.org/article/russian-corruption-the-kremlin-fails-to-tackle-its-biggest-problem.

[CR40] Rochliz M (2014). Corporate raiding and the role of the state in Russia. Post-Soviet Affairs.

[CR41] Rochliz M, Kazun A, Yakovlev A (2020). Property rights in Russia after 2009: From business capture to centralized corruption?. Post-Soviet Affairs.

[CR42] Rosstat (Federal State Statistics Service) (2007). Regiony Rossii 2007.

[CR43] Sutela P, Balling M (2009). Russian finance: Drag or booster for future growth?. Current trends in the Russian financial system.

[CR44] TASS. 2016. Shuvalov: Pravitel’stvo podderzhit vse monogoroda, February 11, https://tass.ru (**in Russian**).

[CR45] Titaev, K. 2012. V Rossii uzhe est‘spravedlivaia sudebnaia sistema – arbitrazhnaia, *Vedomosti*. May 17 (**in Russian**).

[CR46] Uskova T (2012). Monogorod: upravlenie razvitiem.

[CR47] Van de Ven WPMM, Van Praag BMS (1981). The demand for deductibles in private health insurance: A probit model with sample selection. Journal of Econometrics.

[CR48] Varese F (2001). The Russian Mafia: Private protection in a new market economy.

[CR49] Viktorov I, Magyar B (2019). Russia’s network state and *Reiderstvo* practices. Stubborn structures.

[CR50] Volkov V (2002). Violent entrepreneurs: The use of force in the making of Russian capitalism.

[CR51] Voluiskaia, M. 2019. Chto takoe monogorod? *AiF.ru (Argumenty i fakty),* October 29. (**in Russian**)

[CR52] World Bank, 2010. *Russian Economic Report.* No. 22, June 16. www.worldbank.org.ru

[CR53] World Bank. 2022. *War in the region: Europe and Central Asia economic update*. Spring 2022.

[CR54] Yakovlev A, Ivanov D (2021). Friendly bureaucrats, formal rules and firms’ investment decisions: Evidence from a survey experiment in Russia. International Journal of Emerging Markets.

[CR55] Zubarevich, N. 2011. Chetyre Rossii. *Vedomosti,* December 30 (**in Russian**).

